# Probing Multivalent Carbohydrate-Protein Interactions With On-Chip Synthesized Glycopeptides Using Different Functionalized Surfaces

**DOI:** 10.3389/fchem.2021.766932

**Published:** 2021-10-26

**Authors:** Alexandra Tsouka, Kassandra Hoetzel, Marco Mende, Jasmin Heidepriem, Grigori Paris, Stephan Eickelmann, Peter H. Seeberger, Bernd Lepenies, Felix F. Loeffler

**Affiliations:** ^1^ Department of Biomolecular Systems, Max Planck Institute of Colloids and Interfaces, Potsdam, Germany; ^2^ Institute of Chemistry and Biochemistry, Freie Universität Berlin, Berlin, Germany; ^3^ Department of System Dynamics and Friction Physics, Institute of Mechanics, Technical University of Berlin, Berlin, Germany; ^4^ Institute for Immunology and Research Center for Emerging Infections and Zoonoses, University of Veterinary Medicine Hannover, Hannover, Germany

**Keywords:** glycopeptides, glycan binding proteins, lectin—carbohydrate interaction, multivalency, surface functionalization

## Abstract

Multivalent ligand–protein interactions are a commonly employed approach by nature in many biological processes. Single glycan–protein interactions are often weak, but their affinity and specificity can be drastically enhanced by engaging multiple binding sites. Microarray technology allows for quick, parallel screening of such interactions. Yet, current glycan microarray methodologies usually neglect defined multivalent presentation. Our laser-based array technology allows for a flexible, cost-efficient, and rapid *in situ* chemical synthesis of peptide scaffolds directly on functionalized glass slides. Using copper(I)-catalyzed azide–alkyne cycloaddition, different monomer sugar azides were attached to the scaffolds, resulting in spatially defined multivalent glycopeptides on the solid support. Studying their interaction with several different lectins showed that not only the spatially defined sugar presentation, but also the surface functionalization and wettability, as well as accessibility and flexibility, play an essential role in such interactions. Therefore, different commercially available functionalized glass slides were equipped with a polyethylene glycol (PEG) linker to demonstrate its effect on glycan–lectin interactions. Moreover, different monomer sugar azides with and without an additional PEG-spacer were attached to the peptide scaffold to increase flexibility and thereby improve binding affinity. A variety of fluorescently labeled lectins were probed, indicating that different lectin–glycan pairs require different surface functionalization and spacers for enhanced binding. This approach allows for rapid screening and evaluation of spacing-, density-, ligand and surface-dependent parameters, to find optimal lectin binders.

## Introduction

Glycan-protein interactions exist in many biological processes, such as protein folding, cell-cell interaction, cell-adhesion, and signaling. Thus, their understanding is of fundamental importance (Varki, 2009). Glycan arrays are considered versatile tools for high-throughput screening of such interactions. Immobilization of glycans on solid support by high-precision robotics can be achieved in multiple ways, (Geissner et al., 2014; Geissner et al., 2019; O’Neil et al., 2018; Purohit et al., 2018; Mende et al., 2019) becoming nowadays a dominant methodology for detection of novel interactions in immunological and biomedical research (Varki, 2009; Gao et al., 2019), as well as drug discovery (Geissner and Seeberger, 2016; Rademacher et al., 2019; Tikhonov et al., 2020).

Glycans play a key role in diseases and virulence (*e.g.,* diabetes, inflammation, cancer, infections), rendering scientists to investigate their structural and functional characteristics (Zhou and Cobb, 2021). Their interaction with other cells, and their recognition by glycan binding proteins (GBPs), so called lectins, triggered the investigation of their binding ability, and molecular mechanism (Raman et al., 2016; Valverde et al., 2020). Individual interactions between glycans and their GBPs are relatively weak (*e.g*., K_d_ values ≈ µM–mM range). The recognition process that nature has evolved to enhance the binding strength and specificity is called multivalency. This effect enables high binding affinities *via* simultaneous recognition of one or several glycans by GBPs, which have multiple and spatially well-defined glycan binding sites (Fasting et al., 2012; Haag, 2015). For a strong multivalent interaction, not only the type(s) of sugar(s), but also their spatial orientation, their accessibility, and the carrier scaffold are important, to achieve optimum distance with the binding pockets of the multivalent receptor.

Despite the importance of multivalency, it is often neglected on the solid support, since the density and the spacing between sugar moieties is difficult to be determined. Therefore, various studies in the last years focused on the optimum glycan presentation, concentration, flexibility, orientation, and density in the array format (Oyelaran et al., 2009; Müller et al., 2016; Kim et al., 2018; Mende et al., 2019; Valles et al., 2019; Di Maio et al., 2021). In addition, a plethora of multivalent glycan scaffolds have been investigated with diverse size and shape to mimic the natural recognition (Cecioni et al., 2015; Delbianco et al., 2016; Redman and Krauss, 2021). Peptide scaffolds have been widely studied due to their simple synthesis *via* solid phase peptide synthesis, (Merrifield, 1963) offering well-defined monodisperse structures. Introduction of sugars on the peptide moieties can be employed using glycosylated amino acids or, in a concerted fashion onto unnatural, azido modified amino acids for specific conjugation (e.g., Click chemistry or Staudinger Ligation) (Specker and Wittmann, 2006; Freichel et al., 2017; Hill et al., 2018; Camaleño de la Calle et al., 2019).

Yet, the application of this approach in the microarray format remains challenging. Fabrication of natural glycoproteins, (Kilcoyne et al., 2012) neoglycopeptides, (Wang et al., 2002) glycodendrimers, (Laigre et al., 2018) DNA-based glycoconjugates, (Hawkes et al., 2019) glycoclusters, (Moni et al., 2009) and glycopolymers (Godula et al., 2009; Zilio et al., 2015) in the microarray format with multivalent presentation require extensive synthetic work prior to the printing onto glass slides. Unfortunately, printing of these compounds on the microarray ties in with solubility and density fluctuations of the material, printing and humidity inconsistencies during coupling, and the microarray surface functionalization (linker) effect, resulting in insufficient coupling and/or poor morphology of the spotted material (Ruprecht et al., 2019; Temme et al., 2019).

Herein, we report our progress in and deeper understanding of our laser-based method for *in-situ* generation of multivalent glycopeptides in the microarray format with controlled glycan spacing and density (Mende et al., 2020). We expanded our technology, making it compatible with different commercially available microarray surfaces, to probe previously inaccessible glycan interactions. Therefore, we first optimized the synthesis on each microarray surface type and we equipped them with an additional linker to investigate its effect on lectin binding. We demonstrate the importance of surface accessibility and wettability on glycan-GBP interactions, enabling us to study a wide range of plant lectins in a high-throughput manner.

## Materials and Methods

### Donor Slide Preparation

Microscope glass slides (Marienfeld Superior, Germany; size 76 × 26 × 1 mm, ground edges, pure white glass) were covered on one side with self-adhesive polyimide foil (Kapton, DuPont, United States, CMC Klebetechnik GmbH, Germany; thickness of polyimide layer approximately 25 μm, thickness of glue layer approximately 45 μm). A thin layer of the transfer material was placed on top of the polyimide foil by spin coating (80 rps, Schaefer Technologie GmbH, Germany; KLM Spin-Coater SCC-200). Two different spin coating solutions were prepared. Pentafluorophenyl (OPfp)-activated 9-fluorenylmethoxycarbonyl (Fmoc) protected l-glycine, (Fmoc-Gly-OPfp) **1** (3.00 mg), was pre-dissolved in dimethylformamide (DMF) (50 µL), while inert polymer matrix (27 mg) (SLEC PLT 7552, Sekisui Chemical GmbH, Germany) was dissolved in dichloromethane (DCM) (450 µL), resulting in the final spin coating solution (500 µL). The non-activated amino acid, Fmoc-propargyl-glycine (Fmoc-Pra-OH) (3 mg) was pre-dissolved in DMF (50 μL), followed by addition of *N,N′*-diisopropylcarbodiimide (DIC) (1.4 µL) and pentafluorophenol (PfpOH) (1.7 mg) consecutively, while the inert polymer matrix (27 mg) was pre-dissolved in DCM (450 μL), forming the desired Fmoc-Pra-OPfp **2** *in situ* (see Supplementary Material).

### Acceptor Slide Preparation

Fmoc-NH-β-Ala-PEGMA-co-MMA glass slides (∼20 nm thick coating, loading of functional groups according to vendor 1 nmol cm^−2^, estimated functional group spacing of 7–10 nm) were acquired from PEPperPRINT GmbH (Germany) and the 3D-Amino glass slides (according to vendor 1–5 nmol cm^−2^) from PolyAn GmbH (Germany). On PolyAn and PEPperPRINT slides, a hydrophilic PEG ((EG)_3_) -based spacer (≈17 Å length) was attached (see Supplementary Material, Section 3.2), before the synthesis of the desired tetrapeptides. In a variant process, PolyAn slides without PEG-spacer were used directly, without prior spacer functionalization.

### Laser Transfer Parameters

For the array synthesis, a spot pitch of 250 μm was used. A laser scanning system with 488 nm wavelength and 120 mW maximum output power was used (Mende et al., 2020), with a laser focus diameter of ∼20 µm. *PEPperPRINT slides:* A laser power of 80 mW and a pulse duration of 6 ms per spot was applied. *PolyAn slides:* A laser power of 60 mW with a pulse duration of 6 ms was applied. The final spot diameter was about 150 μm.

### General Laser-Based Synthesis Process and Synthesis of Tetrapeptide Scaffolds

*General laser-based synthesis process*: The laser transfer and peptide synthesis were conducted as reported previously (Eickelmann et al., 2019; Mende et al., 2020; Paris et al., 2020). The process begins with the preparation of different donor slides (*Donor Slide Preparation*) that are easily prepared by spin-coating a solution of polymer matrix and activated amino acid building block onto polyimide foil (Kapton) bearing glass slides. The polymer and amino acid mixture forms an about 200 nm thin layer on the polyimide. For the patterning process, an amino acid containing donor slide is placed on top of an acceptor slide (*Acceptor Slide Preparation*) and a focused laser (*Laser Transfer Parameters*) transfers solid polymer material spotwise from the donor to the acceptor (one pulse of 6 ms transfers one spot). The laser is absorbed by the polyimide foil, which heats up and expands. Eventually, the expanding polyimide contacts the acceptor slide, causing the transfer of nanometer thin and about 150 µm wide polymer material spots. The transfer is repeated with different donor slides until the desired amino acid pattern is completed. Afterwards, the acceptor slide is placed into an oven at 95°C under nitrogen for several minutes to initiate the coupling reaction. In the oven, the polymer spots “melt” while retaining their shape, enabling the reaction of the building blocks according to the transferred pattern. The activated amino acid building blocks couple to the amino groups on the acceptor slide. Next, the acceptor slide is washed, removing unreacted amino acids and residual polymer. Each amino acid coupling step is repeated three times to increase the coupling yield and to minimize deletion sequences. Then, remaining free amino groups on the acceptor surface are acetylated and the Fmoc protecting groups are removed before the next synthesis cycle. Peptide synthesis steps are repeated, until the final peptide length is reached.

*Synthesis of tetrapeptide scaffolds:* Commercially available slides from PEPperPRINT or PolyAn were used. Before the synthesis of the tetrapeptides, a PEG-based spacer was attached if not indicated otherwise, (see Supplementary Material). PEPperPRINT slides require a spacer due to the high protein resistance of the surface. The first layer of OPfp-activated and Fmoc-protected amino acids was transferred *via* laser transfer, using two different donor slides sequentially to create the desired combinatorial pattern on the acceptor slide. The coupling reaction was accomplished under heat in an oven under nitrogen atmosphere at 95°C for 10 min. Subsequently, the slides were washed with acetone twice, initially for 2 min in an ultrasonic bath, and then for another 2 min in a petri dish on a shaker (450 rpm). Then, slides were dried in a jet of air. The laser transfer of the same amino acid pattern, the coupling, and the acetone washing steps were repeated twice, to increase the coupling efficiency. Each time, a new donor slide was used for every transfer and coupling cycle. Free unreacted amino groups on the slides were acetylated with a capping solution twice for 30 min (see Supplementary Material). The slides were washed with DMF (3 × 5 min), methanol (MeOH) (1 × 2 min), DCM (1 × 1 min), and dried in a jet of air. Deprotection of the terminal Fmoc-groups was achieved for 20 min with Piperidine (see Supplementary Material) on a shaker (450 rpm). The slides were washed with DMF (3 × 5 min), MeOH (1 × 2 min), DCM (1 × 1 min), consecutively, and dried in a jet of air. The whole process was repeated, as needed, for each pattern to synthesize the desired peptides. In the case of terminal amino acids within the peptide chain, the Fmoc removal was accomplished before the acetylation step, capping of the free amino groups.

### Sugar Azides

Each sugar azide **3–7** was obtained according to known literature procedures (see Supplementary Material, Section 2.1). Two sugar azides, **8** and **9**, were obtained from *Conju-Probe*.

### Copper (I)-Catalyzed Alkyne-Azide Cycloaddition (CuAAC)

CuSO_4_ (530 μg, 3.36 μmol, 2.00 equiv) was dissolved in a mixture of dimethyl sulfoxide (DMSO) and water (1:1, 200 μL). Sodium ascorbate (998 μg, 5.04 μmol, 3.00 equiv) was added and the mixture was thoroughly vortexed. The precipitate was centrifuged for 1 min. The remaining solution was passed through a polypropylene syringe filter (0.2 µm polypropylene filter media with polypropylene housing, 25 mm diameter, Whatman, Global Life Sciences Solutions Operations United Kingdom). The sugar azide (1.68 μmol, 1.00 equiv) was dissolved in this solution and then applied on the acceptor surface (c = 8.4 μmol/ml). For the incubation, a 16-well format incubation chamber was used. The prepared solution (200 μL) was poured in one of the wells and then shaken overnight in the dark. The next day, the slide was washed with water three times for 5 min inside the well and one time for 30 min in a petri dish on a shaker (450 rpm). Finally, the slide was dried in a jet of air.

### Plant Lectin Assay

To avoid unspecific binding, the acceptor slides were incubated with a blocking buffer for 40 min (Rockland, United States; blocking buffer for fluorescent western blotting MB-070). Fluorescently labeled plant lectins, *concanavalin A* (*i.e.*, ConA; CF^®^633 ConA, Biotium, Inc., United States) was diluted to 100 μg/ml in lectin buffer (50 mM HEPES, 100 mM NaCl, 1 mM CaCl_2_, 1 mM MnCl_2_, 10% blocking buffer, 0.05% Tween 20, pH 7.5), *ricinus communis* agglutinin I, (RCA-I), *peanut* agglutinin (PNA), *soybean* agglutinin (SBA), *dolichos biflorus* agglutinin (DBA), and *wheat germ* agglutinin (WGA) (Rhodamine labeled, Lectin kit 1, Vector laboratories, United States) were diluted to 10 μg/ml in lectin buffer and incubated for 1 h at room temperature. Subsequently, each stained well was washed with PBS-T (3 × 5 min). Then, the acceptor slide was rinsed with Tris buffer (1 mM Tris-HCl buffer, pH = 7.4) to remove all the remaining salt residues, and dried in a jet of air. Fluorescence scanning was used to detect the lectin binding on the corresponding sugar moieties.

### Fluorescence Scan

All fluorescence scans were carried out in a high-resolution microarray GenePix 4000B scanner. CF^®^ConA labeled glycopeptides were screened with an excitation wavelength of 635 nm and PMT gain of 600. Rhodamine RCA-I, PNA, SBA, DBA, WGA labeled glycopeptides were scanned at an excitation wavelength of 532 nm and PMT gain of 500. Carboxytetramethylrhodamine (TAMRA) labeled tetrapeptides were detected at an excitation wavelength of 532 nm and PMT gain of 400. The laser power was always set to 33% and the pixel resolution to 5 μm. For the analysis of the fluorescence images, the analysis software GenePix Pro 6.0 (Molecular Devices, Sunnyvale/California, United States) was used.

### Analysis of Glycopeptides Regarding Multivalency Effects

For each sugar azide, the reaction was performed in a separate cavity of a 16-well format incubation chamber (PEPperPRINT GmbH, Germany). Each well contained three sets of quadruplicates of the same single sugar azide and tetrapeptide, giving twelve glycopeptide replicas of each synthesized structure. The median of the fluorescence intensity of the scanned area was determined with the microarray analysis software GenePix Pro 6.0. For the analysis, the mean value of the twelve spot medians was calculated. Spots (*i.e.,* outlier/artifacts) with more than 40% standard deviation from the mean were excluded from calculations.

## Results and Discussion

**FIGURE 1:**
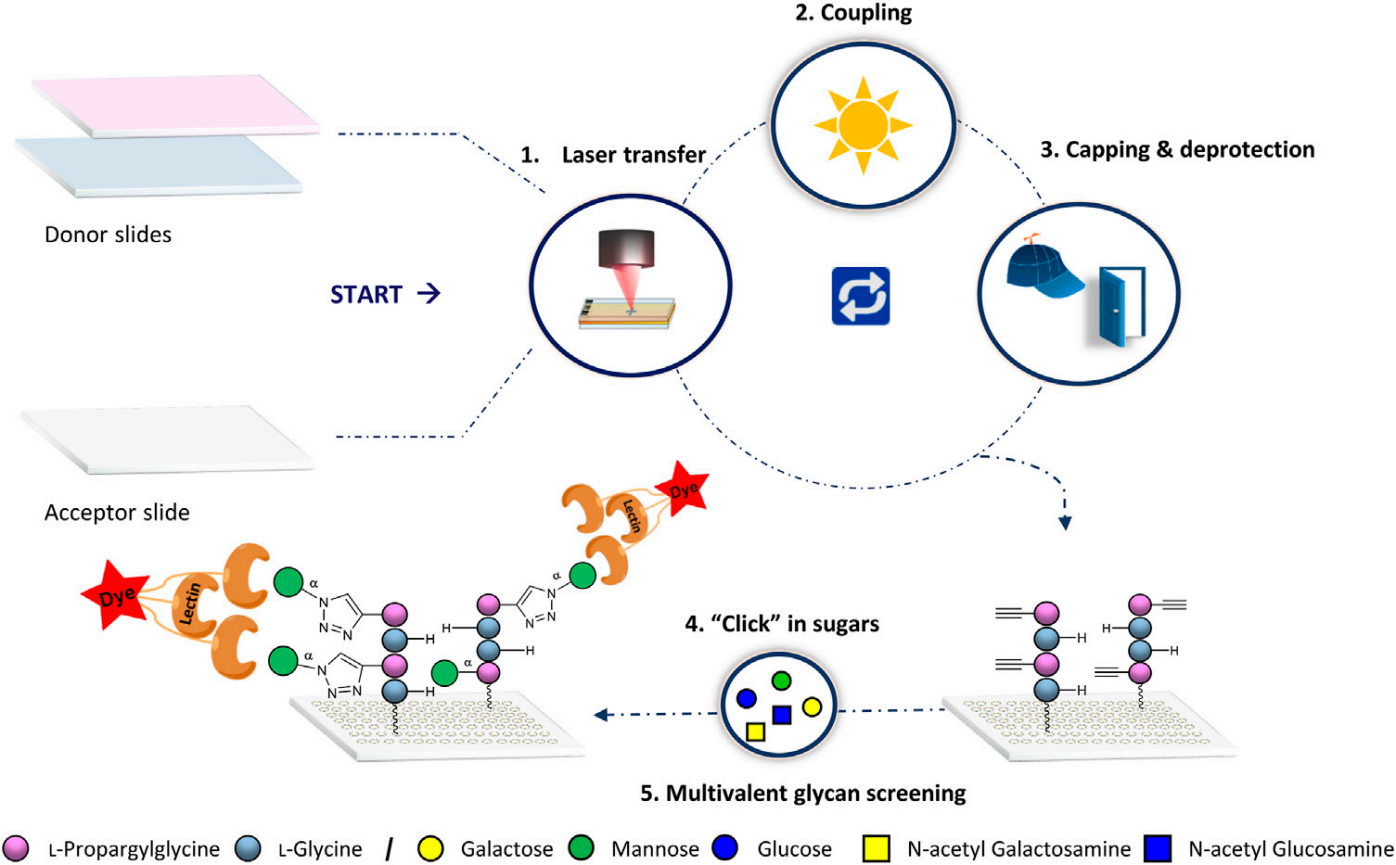
General synthetic approach for glycopeptide generation. Synthesis consists of the 1) laser transfer process, 2) coupling of the amino acids on defined spots, 3) capping to mask unreacted amine groups with subsequent deprotection of the Fmoc protecting group for the next coupling step, and 4) attachment of the sugar azides on the peptide scaffolds by CuAAC. Finally, 5) binding of lectins is screened for multivalent interactions.

We applied our laser transfer technology to generate peptide scaffolds directly in the array format.(Loeffler et al., 2016; Eickelmann et al., 2019; Mende et al., 2020) Therefore, different donor slides were prepared, containing alkyne-functionalized l-propargylglycine (Pra) or l–Glycine (Gly) amino acid building blocks. These donor slides were placed on top of a functionalized acceptor slide and a laser precisely transferred the building blocks in desired patterns. Next, the amino acid pattern was coupled in an oven to the acceptor slide, the surface was washed, capped and Fmoc deprotected. Repeating these *in-situ* solid phase synthesis steps, peptides were generated in the array format on the acceptor. Finally, copper (I)-catalyzed alkyne-azide cycloaddition (CuAAC) was used to attach different azido-functionalized glycan monomers to the alkyne groups of the peptide scaffolds (Figure 1).

For our work, we aimed to employ different commercially available amine functionalized acceptor slides from different suppliers to determine the influence of the surface functionalization on glycan binding events. Hydrophobicity and sterical hindrance of the acceptor surface functionalization may lead to lower accessibility of the glycans. Therefore, we had to find new process conditions for the synthesis of the peptides on the different functionalized slides. Then, we studied the interactions of the fluorescently labeled lectins on these substrates and analyzed with fluorescence scanning. To compare high and low- affinity glycan-GBP interactions, we chose to probe the plant lectins *concanavalin A* (ConA; tetramer), *ricinus communis* agglutinin I (RCA-I; tetramer with only two Gal-specific subunits) (Wittmann and Pieters, 2013)), *peanut* agglutinin (PNA; tetramer), *soybean* agglutinin (SBA; tetramer), *dolichos biflorus* agglutinin (DBA; tetramer), and *wheat germ* agglutinin (WGA; dimer) with their corresponding glycans under the same conditions. Furthermore, we screened the CLR-Fc fused C-type lectins mLangerin, mMincle, and mMGL-1 (Maglinao et al., 2014; Artigas et al., 2017; Mayer et al., 2017; Valverde et al., 2020). However, since we did not observe any binding of these three lectins, details are only discussed in the Supplementary Material (Section 8).

### Synthesis of Glycopeptides

**FIGURE 2:**
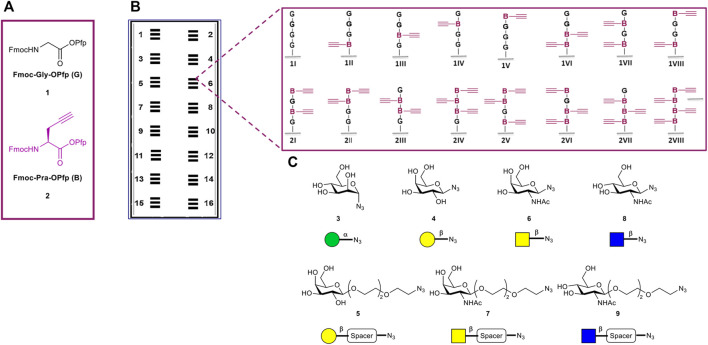
Overview of tetramer peptide scaffolds and sugar azides. **(A)** The two amino acids used for the synthesis of the desired scaffolds. **(B)** Representation of the 16 synthesized microarrays per glass slide, each array containing three copies of the sixteen different tetramers in quadruplicate spots (*n* = 3 × 4 = 12 spots of each structure per microarray). **(C)** Collection of sugar azides for the generation of the glycopeptides.

All sixteen possible variants of the peptide tetramers, containing the two derivatives Fmoc-Gly-OPfp **1** and Fmoc-Pra-OPfp **2**, were synthesized in the microarray format (Figure 2). Amine functionalized glass slides from PEPperPRINT (PPP) were used with prior functionalization with a PEG-based spacer (Stadler et al., 2008). 3D-amino glass slides from PolyAn were either used with or without prior PEG-spacer functionalization. Before the synthesis, we optimized the transfer and coupling conditions for each solid support (see Supplementary Material, Section 4). Subsequently, a pre-patterning of all acceptor slides was performed with two glycines **1**, to further increase the distance between the tetrapeptides and the solid support and, thereby, the accessibility of the glycopeptides. After Fmoc deprotection of the N-terminus, the free amino groups were used for peptide synthesis. Two donor slides were employed to synthesize the sixteen tetrapeptide combinations, Fmoc-Gly-OPfp **1** (**G**) and Fmoc-Pra-OPfp **2** (**B**) Figure 2A (conventional synthesis from C-terminus to N-terminus, *e.g.,* N-GBGB-C, 1VII). Coupling and laser transfer of each amino acid layer was repeated three times to achieve high coupling efficiency and prevent deletion sequences while growing the chains. Coupling of the amino acids was conducted in an oven under nitrogen gas atmosphere at 95°C, resulting in three sets of quadruplicates on one array (*n* = 12 spots; binding intensity is calculated as the mean of the 12 spot replica) (Figure 2B). Quality control of the three synthesized arrays was carried out *via* clicking a TAMRA azide dye to the scaffolds and analyzing the fluorescence intensity. On the PEPperPRINT slides, a rather constant fluorescence intensity was observed, indicating a quenching effect for higher valencies, as reported previously (Mende et al., 2020). Comparing the results of the two PolyAn slides with and without PEG-spacer, also some quenching could be observed (see Supplementary Material for more details, Section 5).

### CuAAC of the Sugars for Glycopeptide Synthesis

The copper(I)-catalyzed alkyne-azide cycloaddition (CuAAC) has been widely used in the last years for the synthesis of glycoconjugates on solid support (Freichel et al., 2017; Hill et al., 2018; Camaleño de la Calle et al., 2019; Mende et al., 2020). Herein, we used this approach to attach the following collection of sugar azide monomers to our synthesized peptide scaffolds: α-mannose (α-Man) azide **3**, β-galactose (β-Gal) azide **4**, β-galactose PEG3-spacer (β-Gal-PEG3) azide **5**, *N*-acetyl-β-galactosamine azide (β-GalNAc) **6**, *N*-acetyl-β-galactosamine PEG3-spacer (β-GalNAc-PEG3) azide **7**, *N*-acetyl-β-glucosamine (β-GlcNAc) **8**, and *N*-acetyl-β-glucosamine PEG3-spacer (β-GlcNAc-PEG3) azide **9**. The sugar azides **3**–**6** and **8** were synthesized based on known experimental procedures from their corresponding unmodified building blocks, while compounds **7** and **9** were commercially acquired (Figure 2C, see Supplementary Material). Each CuAAC reaction with individual sugars was performed in a separate well, reacting all peptide scaffold spots (*n* = 12) of one array with on sugar.

### Glycan-GBP Assays and Fluorescence Evaluation

**FIGURE 3:**
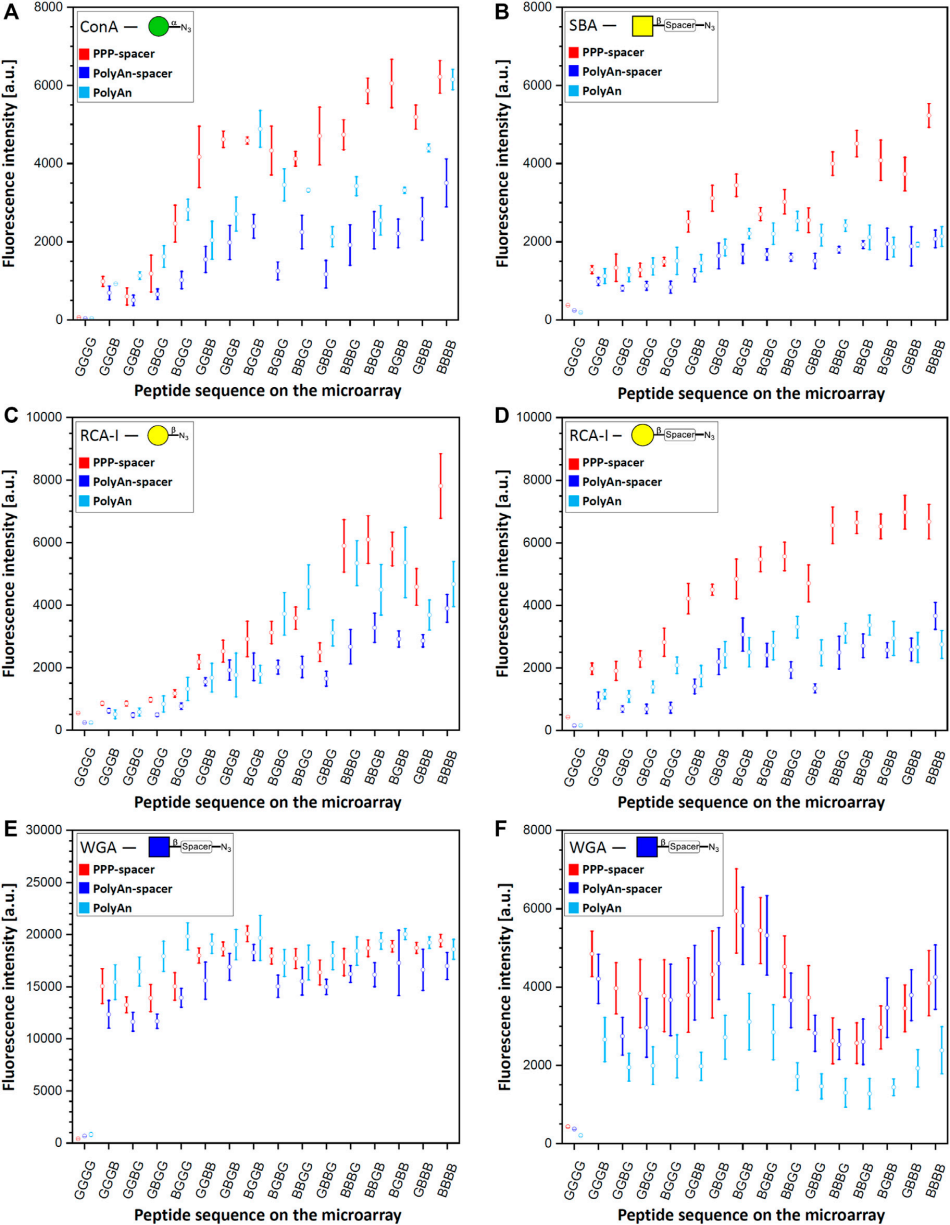
Fluorescence staining intensities of respective sugar azides **3**, **4**, **5**, **7** and **9** with their corresponding lectins: **(A)** α-Man azide **3** with ConA (CF^®^633 labeled, 100 μg/ml concentration), **(B)** β-GalNAc-PEG3 azide **7** with SBA (rhodamine labeled, 10 μg/ml concentration) **(C)** β-Gal azide **4** with RCA-I (rhodamine labeled, 10 μg/ml concentration), **(D)** β-Gal-PEG3 azide **5** with RCA-I (rhodamine labeled, 10 μg/ml concentration), **(E)** β-GlcNAc-PEG3azide **9** with WGA (rhodamine labeled) with 10 μg/ml concentration, and **(F)** 0.2 μg/ml concentration on PEPperPRINT slides with PEG-spacer (PPP-spacer; red), and on PolyAn functionalized slides with (dark blue) and without PEG-spacer (light blue).

After the generation of the glycopeptides on the differently functionalized acceptor slides, we probed the synthesized structures with their corresponding fluorescently labeled lectins (Figure 3). Tetrapeptides, carrying the α-Man azide **3** were incubated with ConA (100 μg/ml, Figure 3A). Structures with β-Gal azide **4**, and β-Gal-PEG3 azide **5**, were probed with fluorescently labeled RCA-I, (Figure 3C, D) and PNA (10 μg/ml, Supplementary Material, Section 7.2). Tetrapeptides with attached β-GalNAc azide **6** and β-GalNAc-PEG3 azide **7** were incubated with DBA and SBA (10 μg/ml) (see Supplementary Material, Sections 7.3 and 7.4), while scaffolds with β-GlcNAc **8** (see Supplementary Material, Section 7.6) and β-GlcNAc-PEG3 azide **9** were probed with WGA (Figure 3E, F) (10 μg/ml). Since we observed an intensity plateau with WGA already for divalent structures, which was different from all other lectins, a 50-fold decreased WGA concentration (0.2 μg/ml) was screened additionally. We analyzed the spacing, density, and ligand dependent binding, and we could confirm that protein binding is surface dependent. In the case of the multivalent glycan-GBP interactions, similar intensity trends were observed for all used lectins on the microarrays (except for WGA, Figure 3E, F), with an increase in binding with an increasing number of sugars on the peptide backbone. Structures with only one attached sugar moiety, *e.g*., BGGG, GGBG, GBGG, GGGB, showed structure dependent binding, with higher intensity for the N-terminal propargylglycine on all used slides. This could be explained by the higher distance between the sugar and the surface, making it more accessible. The tetra-glycine scaffold (GGGG) was considered as the background control.

In terms of slide functionalization, for all detected interactions, the fluorescence intensities were higher on the PEPperPRINT slides (apart from WGA and DBA). Between the two differently functionalized PolyAn slides, some structure and lectin dependent binding differences were observed.

#### Binding Studies on PEPperPRINT Slides

On PEPperPRINT slides, which were always equipped with the PEG-spacer, the binding of ConA to α-Man azide **3** increased exponentially with linear increase in the number of sugar moieties Our divalent scaffolds show an up to 10-fold increase in fluorescence signals in comparison to the monovalent ones, while the trivalent show an up to 20-fold, and the tetravalent is in the range of the trivalent system without significant change on the binding ability (Figure 3A). This trend agrees with our previous data (Mende et al., 2020). However, with the here introduced optimized synthetic conditions (see Supplementary Material, Section 4), the observed intensities are one order of magnitude higher with the same assay protocol. On the same acceptor slides, we screened multiple sugar monomers with and without PEG-spacer at the anomeric center. Binding of PNA, DBA, and SBA to β-Gal azide **4**, β-Gal-PEG3 azide **5**, and β-GalNAc azide **6**, respectively, was not observed. Notably, multivalent binding was only detected for SBA to the β-GalNAc-PEG3 azide **7** (Figure 3B). The enhanced flexibility between the anomeric position and the azide moiety given from the spacer allows the SBA to bind to the more flexible β-GalNAc-PEG3 azide **7**, but not to the β-GalNAc azide **6**. The fluorescence intensities of SBA on PEPperPRINT slides follow the same binding trend as ConA, but the binding to the tetravalent *vs*. the monovalent structures only increases about 6-fold. Despite the fact that ConA and SBA differ in their sugar specificity, both have similar orientation of binding sites and ligand recognition mechanism (Sinha et al., 2005). In contrast, RCA-I binds to both, β-Gal azide **4** and β-Gal-PEG3 azide **5** (Figure 3C, D). Interestingly, for the more flexible β-Gal-PEG3 azide **5**, the binding intensities of RCA-I are already at least 4-fold higher for the monovalent structures in comparison to the β-Gal azide **4**. Again, the PEG-spacer increases the flexibility of the sugar moiety and increases the distance to the triazole ring, making it more accessible for the lectin. Thus, the multivalent effect is much more pronounced for the β-Gal azide **4** than the β-Gal-PEG3 azide **5**, while the tetravalent structures from both reach a similar maximum (*i.e.,* saturation) intensity at our tested lectin concentration.

Similarly, WGA binds stronger to β-GlcNAc-PEG3-azide **9** structures (Figure 3E, F) than to β-GlcNAc azide **8** (see Supplementary Material, Section 7.6). All other lectins we studied are tetramers, WGA is the only dimer and its binding was markedly different to all other lectin binding experiments. The intensity is already high for the monovalent structures and seems to reach a plateau/saturation for divalent structures. To assess the potential impact of a lower lectin concentration, we also tested a 50-fold decreased WGA concentration (Figure 3F). While, as expected, with lower concentration the total intensity was lower, a very similar trend as in the higher concentration could still be observed. Yet, a somewhat decreased intensity for trivalent structures was apparent, which seems to be a density or spacing effect. Notably, for WGA, the monovalent structure GGGB has a stronger binding (sugar is close to the surface), while generally for all other lectins, the monovalent structure BGGG (sugar is furthest away from the surface) gives the highest intensity.

Our studies show a spacing dependent binding for the divalent systems. Higher intensities for ConA (to α-Man azide **3**), and RCA-I (to β-Gal-PEG3 azide **5**) are attained for non-adjacent divalent structures (GBGB, BGGB, BGBG). A similar effect is observed for trivalent binders: the intermediate glycine (BGBB, BBGB) increases the binding for ConA, SBA, and RCA-I (β-Gal azide **4**) in comparison to structures with terminal glycines (BBBG, GBBB). For RCA-I, the more flexible β-Gal-PEG3 azide **5** shows a generally higher binding, but especially on the trivalent system with the C-terminal glycine (BBBG). In the case of WGA, the divalent scaffolds with two neighboring Pra moieties (BBGG, GGBB, GBBG) give less binding, while stronger binding is obtained on non-neighboring Pra scaffolds (GBGB, BGBG, BGGB).

#### Binding Studies on PolyAn Slides

Next, we investigated the impact of a different commercial substrate on the binding of the lectins. Thus, we functionalized the more hydrophilic PolyAn slides with the same PEG-spacer (see Supplementary Material, Section 3.2.). We measured the hydrophobicity of all used slides (PEPperPRINT and PolyAn) with and without PEG-spacer, showing that the hydrophilic character of the PoyAn slides does not change after the attachment of the PEG-spacer (Supplementary Material, Section 6). Comparing the PolyAn to the PEPperPRINT slides, generally similar interactions were detected, while some distinct differences for multivalency, sugar density, and spacing could be observed. The binding ability of ConA on PolyAn slides bearing the PEG-spacer decreased by a factor of 2 compared to the intensities observed on PEPperPRINT slides (Figure 3A). This trend was observed for almost all other lectin interactions. In the case of SBA (Figure 3B), the PolyAn slide surface seems to prevent a multivalent effect (*i.e.,* only linear intensity increase), at least for this lectin concentration. For RCA-I (Figure 3C, D), the PolyAn slide without PEG-spacer showed a similar trend as the PEPperPRINT slide for the β-Gal azide **4**. For β-Gal-PEG3 azide **5**, again, both PolyAn slides showed a similar trend to the PEPperPRINT slide, but with a much weaker multivalent effect and a generally 2- to 3-fold lower intensity. In case of WGA with β-GlcNAc-PEG3 azide **9** (Figure 3E, F), the PolyAn surface without PEG showed a generally higher intensity in the assay with high concentration. For the low concentration WGA assay, PolyAn showed a lower intensity, but still the same trend. Similar binding behavior was also observed for β-GlcNAc azide **8** with WGA (see Supplementary Material, Section 7.6).

Interestingly, only on the PolyAn surfaces, DBA showed a weak binding to β-GalNAc azide **6** and β-GalNAc-PEG3-azide **7** (see Supplementary Material, Section 7.3). However, in this case, we also observed a high background signal for the GGGG control, which is a hydrophobic structure. In the future, it should be further investigated, whether a more hydrophobic alkyl linker (instead of PEG) on the surface can increase this binding, since DBA is known to have a hydrophobic adenine-binding site in addition to the carbohydrate recognition domain (Hamelryck et al., 1999).

As reported before with the PEPperPRINT slides, no binding could be identified for SBA and PNA with β-Gal azide **4**, β-Gal-PEG3 azide **5**, and β-GalNAc azide **6** on PolyAn slides (see Supplementary Material, Sections 7.2, 7.4).

Structure dependent binding was also observed between the different lectins on PolyAn slides. Structures with same theoretical spacing (GBGB and BGBG) do not show the same binding intensities. The strongest binding for WGA on PolyAn slides was detected for the divalent structure BGGB, especially for the lower lectin concentration. Thus, and because the binding sites of WGA are very close to each other (see Conclusion), it indicates cross-linking and chelating binding mode (*i.e.,* two binding sites of WGA bind to one structure). Remarkably reduced binding of WGA was detected on the tri- and tetravalent structures on all substrates compared to the divalent structures, which might be caused by sterical hindrance.

## Conclusion

We describe a flexible and cost-efficient method for the synthesis of defined multivalent glycopeptide arrays. On each microarray, 16 different tetrapeptides were generated *in situ* by our laser-based technology and seven different azido sugar monomers were attached by CuAAC (resulting in a total of 112 different structures on three different surfaces). To study the impact of different commercial surfaces functionalized with different linkers, we first optimized the solid-phase synthesis conditions (amino acid concentration, lasing parameters, coupling time) for different commercial microarray substrates. These optimizations improved the signal-to-noise ratios for our model lectin ConA by one order of magnitude, and helped to expand the applications for our synthesis platform to include weakly binding lectins (*e.g.,* DBA).

Lectin binding depends on spacing, density, surface functionalization, and concentration. PEG-functionalized PEPperPRINT slides provided generally higher signal intensities than PolyAn slides, with the exception of DBA. Lower binding intensities on PolyAn slides equipped with the PEG-spacer indicate that lectin binding decreases under very hydrophilic conditions for the majority of lectins. For a better understanding, we experimentally determined the (water) contact angle of the different surfaces. PEPperPRINT slides are more hydrophobic, while PolyAn slides maintained their hydrophilic character even after the attachment of a PEG-spacer.

Most lectins showed a multivalent binding effect that mainly depends on the valency with exception of the WGA binding assay. A saturation of binding intensity for divalent structures was detected on all microarrays due to the chelating binding mode, leading to cross-linking. Yet, no binding was observed for PNA and DBA on PEPperPRINT slides with simple sugar moieties, while weak interaction was obtained on PolyAn slides with DBA. Spacing of the synthetic scaffolds may not fit the binding sites of most lectins, the selection of sugars was not optimal, and the triazole ring might cause sterical problems. Future investigations will require screening of different mono- and disaccharides, such as lactose and the T-antigen with PNA. In case of DBA, an α-N-acetyl galactosamine residue should offer a much higher binding ability than the β-N-acetyl galactosamine residue. Additionally, longer peptide scaffolds should be synthesized, as well as longer linkers (*e.g.,* PEG5) should be introduced between the anomeric position and the peptide backbone, to increase the size and the flexibility of the synthesized structures.

We were unable to detect any binding between the C-type lectins mLangerin, mMGL-1, and mMincle with their corresponding sugar monomers (see Supplementary Material, Section 8). Interestingly, Di Maio *et al.* very recently reported a microarray assay with multivalent display of mono- and dimannose, where other C-type lectins (DC-SIGNR ECD, trivalent Langerin ECD, monomeric Dectin-2 ECD) were screened. These lectins selectively and strongly bind to Man-α1,2Man, but almost no binding for α-Man monomer was reported (Di Maio et al., 2021). Future screening of disaccharides such as Man-α1,2Man with high valency and staining with directly fluorescently labeled lectins may provide more information on these lectins.

Notably, on PolyAn slides with and without spacer, most lectins showed a more linear (less multivalent) increase in binding with increasing numbers of sugar PEG3 azides. For the less flexible sugar azides without PEG3, typical multivalent trends could be observed.

The molecular spacing of the sugars on the tetrapeptides had a similar impact on ConA, SBA, and RCA-I. Scaffolds with the same theoretical spacing, such as GBGB and BGBG, showed different binding strengths with the latter typically showing a stronger binding strength. Similarly, divalent structures with larger spacing (BGGB) showed stronger binding than the more adjacent scaffolds (*e.g.,* GBBG).

To our knowledge, this work is the first, showing the synthesis of glycopeptides with defined valencies and spacing *in situ* on different commercially available microarrays to investigate the effect of substrate functionalization. Our technology relies on readily available compounds (Eickelmann et al., 2019) and can be fully automated (Paris et al., 2019). This enables us to screen a diverse collection of glycopeptides with their corresponding lectins. We believe that by using other propargyl amino acids in our process in the future, we should be able to find ideal multivalent glycopeptide binders for different lectins. However, the microarray substrate functionalization plays an important role for glycan-GBP interaction studies and has to be thoroughly considered.

## Data Availability

The original contributions presented in the study are included in the article/Supplementary Material, further inquiries can be directed to the corresponding author.
