# Supplementary material for: Probing Multivalent Carbohydrate-Protein Interactions With On-Chip Synthesized Glycopeptides Using Different Functionalized Surfaces
Source: Front Chem. 2021 Oct 26;9:766932. doi: 10.3389/fchem.2021.766932 (PMC8589469; doi:10.3389/fchem.2021.766932)
Supplement: Supplementary file 1 [file DataSheet1.pdf]

Supplementary Material

Probing multivalent carbohydrate-protein interactions with on-chip synthesized glycopeptides using different functionalized surfaces

Alexandra Tsouka[1,2], Kassandra Hoetzel[1], Marco Mende[1], Jasmin Heidepriem[1,2], Grigori Paris[1,3], Stephan Eickelmann[1], Peter H. Seeberger[1,2], Bernd Lepenies[4], Felix F. Loeffler[1]*

[1]Department of Biomolecular Systems, Max Planck Institute of Colloids and Interfaces, Potsdam, Germany

[2]Institute of Chemistry and Biochemistry, Freie Universität Berlin, Berlin, Germany

[3]Department of System Dynamics and Friction Physics, Institute of Mechanics, Technical University of Berlin, Berlin, Germany

[4]Institute for Immunology and Research Center for Emerging Infections and Zoonoses, University of Veterinary Medicine Hannover, Hannover, Germany

***Correspondence:**

Felix F. Löffler

felix.loeffler@mpikg.mpg.de

Index

1 General information 3

1.1 Preparative work 3

1.2 Nuclear magnetic resonance spectroscopy (NMR) 3

1.3 Mass spectrometry (HRMS) 4

1.4 High-performance liquid chromatography (HPLC) 4

1.5 pH measurements 4

1.6 Laser transfer system 5
1.7 Fluorescence scanning 5
1.8 Contact angle measurements 5
1.9 Preparation of solutions 5
2 Experimental Procedures 6
2.1 Synthesis of the sugar azides 6
3 Laser transfer method 12
3.1 Donor slide preparation 12
3.2 Acceptor slide preparation 13
3.2.1 Acceptor slides without spacer 14
3.2.2 Acceptor slides with spacer 14
4 Parameter optimization for the synthesis of tetrapeptides 16
4.1 PEPperPRINT slide optimization 16
4.2 PolyAn slide optimization 18
5 Quality control of synthesized microarrays 20
6 Contact angle measurements 21
7 Binding assays of plant lectins 23
7.1 Concanavalin A (ConA) 23
7.2 Peanut agglutinin (PNA) binding assay 24
7.3 Dolichos biflorus Agglutinin (DBA) binding assay 25
7.4 Soybean agglutinin (SBA) binding assay 26
7.5 Ricinus communis agglutinin I (RCA-I) binding assay 27
7.6 Wheat germ agglutinin (WGA) binding assay 28
8 Binding assay of C-type lectins 29
9 Fluorescence scan images 31
10 Supporting Tables 40
11 References 41

1 General information

1.1 Preparative work

The starting materials, applied solvents, deuterated solvents (99.5 atom% D), and chemicals were purchased from common suppliers such as *Sigma-Aldrich, Alfa Aesar, Tokio Chemical Industry (TCI), Thermo Fischer Scientific, Acros Organics, Iris Biotech, Merck, BroadPharm* and used without further purification. 2-{2-[2-Azidoethoxy]ethoxy}ethyl 2-acetamido-2-deoxy-β-D-glucopyranoside (β-D-GlcNAc-PEG3-azide) and 2-{2-[2-Azidoethoxy]ethoxy}ethyl 2-acetamido-2-deoxy-β-D-galactopyranoside (β-D-GalNAc-PEG3-azide) were obtained from *Conju-Probe*. Lectin Kit I, Rhodamine labeled (RLK-2200), containing: *Concanavalin A* (ConA), *Dolichos Biflorus Agglutinin* (DBA), *Peanut Agglutinin* (PNA), *Ricinus Communis Agglutinin I* (RCA-I/RCA-120), *Soybean Agglutinin* (SBA), *Ulex Europaeus Agglutinin I* (UEA I), and *Wheat Germ Agglutinin* (WGA) (2 mg/mL) was purchased from *Vector laboratories Burlingame*, USA, CF®633 ConA (1 mg/mL) from *Biotium, Inc.*, USA. Anhydrous-solvents were dried on a Phoenix SDS-Flame-Proof Cabinet Mounted, JC-Meyer solvent purification system and stored in glass bottles over freshly activated 3Å molecular sieves. All reactions containing air- and moisture-sensitive compounds were performed under argon atmosphere using oven-dried glassware, applying normal Schlenk-techniques. Liquids were added via steel cannulas and solids were added directly in powdered forms. Reactions were carried out at room temperature (rt), if not indicated otherwise. For low reaction temperatures, flat dewars were used with ice/water or isopropanol/dry ice mixtures. The solvents were removed at 40 °C with a rotary evaporator under reduced pressure. For solvent mixtures, each solvent was measured volumetrically. If not mentioned otherwise, saturated, aqueous solutions of inorganic salts were used. Thin layer chromatography (TLC) using silica gel coated aluminium plates (*MACHEREY-NAGEL*, pre-coated TLC sheets ALUGRAM® Xtra SIL G/UV_{254} or *Merck*, pre-coated TLC sheets 60 F_{254}) was applied to monitor reactions. Detection was performed either with UV light (λ = 254 nm) or by immersing the TLC plates in "Seebach staining solution" (mixture of phosphomolybdic acid hydrate, cerium(IV) sulfate tetrahydrate, sulfuric acid and water). Purification of the nonpolar products was done *via* flash chromatography using *MACHEREY-NAGEL* silica gel 60 (0.040 × 0.063 mm) and quartz sand and very polar compounds were purified by *MACHEREY-NAGEL* silica gel, Chromafix® C_{18} ec cartridge. Eluents were used directly in *p.a.*, HPLC quality or distilled.

1.1 Nuclear magnetic resonance spectroscopy (NMR)

The spectra were recorded on either an Agilent 400-MR (400 MHz) or a Bruker Ascend 400 (400 MHz) spectrometer. Chemical shifts δ are reported in ppm and are adjusted to internal standards of the residual proton signal of the deuterated solvent (D_2O: 4.79 ppm for ^{1}H, MeOD: 3.31 ppm for ^{1}H and 49.00 ppm for ^{13}C spectra). The spectra were measured at room temperature. Having symmetrical signals, the center of the signal is given and for multiplets the area. The following characterization was used: s = singlet, sbr = singlet broad, d = doublet, t = triplet, q = quartet, m = multiplet or combinations like dd = doublet of doublet or dt = doublet of triplet and mc = centered multiplet. Coupling constants (J) are given in Hz. The spectra were evaluated according to 1st order. For ^{1}H NMR spectra, the correlation of the signals was done according to the multiplicities.

1.2 Mass spectrometry (HRMS)

High-resolution mass spectrometry (HRMS) was performed on a Waters Xevo G2-XS QTof device using ESI (electrospray ionization) as the ionization method. The abbreviation $[M+Na]^+$ refers to the product–sodium adduct. ESI mass spectra were run on IonSpec Ultima instruments.

1.3 High-performance liquid chromatography (HPLC)

To identify the purity of the crude used products, analytical HPLC (Agilent 1200 Series spectrometer) was used:

Method A: Synergi Hydro RP18 column, 250 x 4.6 mm, flow rate of 1 mL/min with H_2O (0.1% formic acid) as eluents [isocratic (5 min), linear gradient to 35% ACN (35 min), linear gradient to 100% ACN (5 min)].

Method B: Synergi Hydro RP18 column, 250 x 4.6 mm, flow rate of 1 mL/min with 5% ACN in H_2O (0.1% formic acid) as eluents [isocratic (5 min), linear gradient to 100% ACN (35 min), linear gradient to 100% ACN (5 min)].

Method C: Synergi Hydro RP18 column, 250 x 4.6 mm, flow rate of 1 mL/min with H_2O (0.1% formic acid) as eluents [isocratic (5 min), linear gradient to 20% ACN (35 min), linear gradient to 100% ACN (5 min)].

1.4 pH measurements

pH was determined using a FiveEasy pH/mV meter F20, equipped with a plastic pH electrode LE438 from Mettler Toledo.

1.5 Laser transfer system

Our laser system consists of a 200 mW TOPTICA iBeam smart 488-S laser with a wavelength of 488 nm (TOPTICA Photonics, AG, Gräfelfing/Bayern, Germany), which is passed through a 1:10 beam expander and a Racoon 11 laser scanning system (ARGES GmbH, Wackersdorf/Bayern, Germany), equipped with an f-Theta lens (S4LFT5110/322, Sill Optics GmbH, Wendelstein/Bayern, Germany). High quality laser transfer with reproducible results at various positions is achieved with scanning the laser beam in a 66 mm × 66 mm plane. The acceptor slide in the lasing areas is aligned with three mechanical springs and a vacuum mechanism. (Mende et al., 2020)

1.6 Fluorescence scanning

A Molecular Devices microarray scanner, GenePix 4000B, San Jose, USA, was used for the analysis of all arrays. The detection wavelength was $\lambda = 523$ nm (for TAMRA fluorophore and Rhodamine RCA-I, PNA, SBA, DBA, WGA), with PMT gain 400 and 500 respectively, or $\lambda = 635$ nm (for CF®633 ConA), with PMT gain 600. The laser power was 33% for every measurement and the pixel size was 5 µm for high-resolution scans.

1.7 Contact angle measurements

The contact angle measurements were performed in an in-house build setup (Karpitschka et al., 2017). It consists of a macroscopic top view, which is homogeneously illuminated with a green LED, and a telecentric side view illuminated *via* a pin hole. An environmental chamber allows controlling the vapor saturation. All measurements were performed at ambient conditions.

1.8 Preparation of solutions

- **Fmoc-deprotection:** A solution of 20% piperidine in dimethylformamide (DMF) (v/v) was prepared.
- **Capping:** A solution of 10% acetic anhydride (Ac_2O) and 20% *N*,*N*-diisopropylethylamine (DIPEA) in 70% anhydrous DMF (v/v) was used.
- **Washing steps:** DMF (3 × 10 mL, 3 min each), methanol (MeOH) (1 × 10 mL, 2 min), and dichloromethane (DCM) (1 × 10 mL, 1 min).

2 Experimental Procedures

2.1 Synthesis of the sugar azides

Synthesis of α-D-mannopyranosyl azide (3)

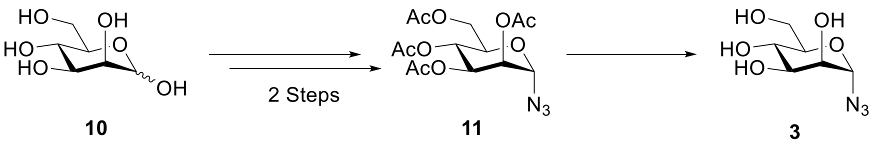

Molecule **3** was synthesized according to the references.(Kang et al., 2015; Mende et al., 2020)

2,3,4,6-Tetra-*O*-acetyl-α-D-mannopyranosyl azide (**11**) (320 mg, 2.71 mmol, 1.00 Equiv.) was dissolved in 5 mL of anhydrous methanol under argon atmosphere and 0.5 M sodium methoxide solution in methanol (0.58 mL) was added. The solution was stirred overnight at room temperature and then neutralized by Amberlite IR-120 H^+. The mixture was filtered over a pad of Celite and the solvent was removed under reduced pressure. The crude product was obtained as an off-white solid in 99% yield (176 mg, 0.86 mmol) and used without further purification.

^{1}H NMR (400 MHz, D_2O): δ = 3.47 – 3.54 (m, 1H, *H*-4), 3.58 – 3.68 (m, 3H, *H*-3, *H*-5, *H*-6β), 3.73 (dd, *J* = 3.3, 1.9 Hz, 1H, *H*-2), 3.75 – 3.81 (m, 1H, *H*-6α), 5.32 (d, *J* = 1.9 Hz, 1H, *H*-1) ppm. **^{13}C NMR** (101 MHz, D_2O): δ = 60.7, 66.3, 69.6, 69.7, 74.5, 89.6 ppm. – HRMS (ESI): calcd for $C_6H_{11}N_3O_5$ Na $[M+Na]^+$: 228.0590; found: 228.0585.

The analytical data agree with the literature.(Kang et al., 2015; Mende et al., 2020)

RP-HPLC (ELSD trace, Method A, t_R= 8.9 min)

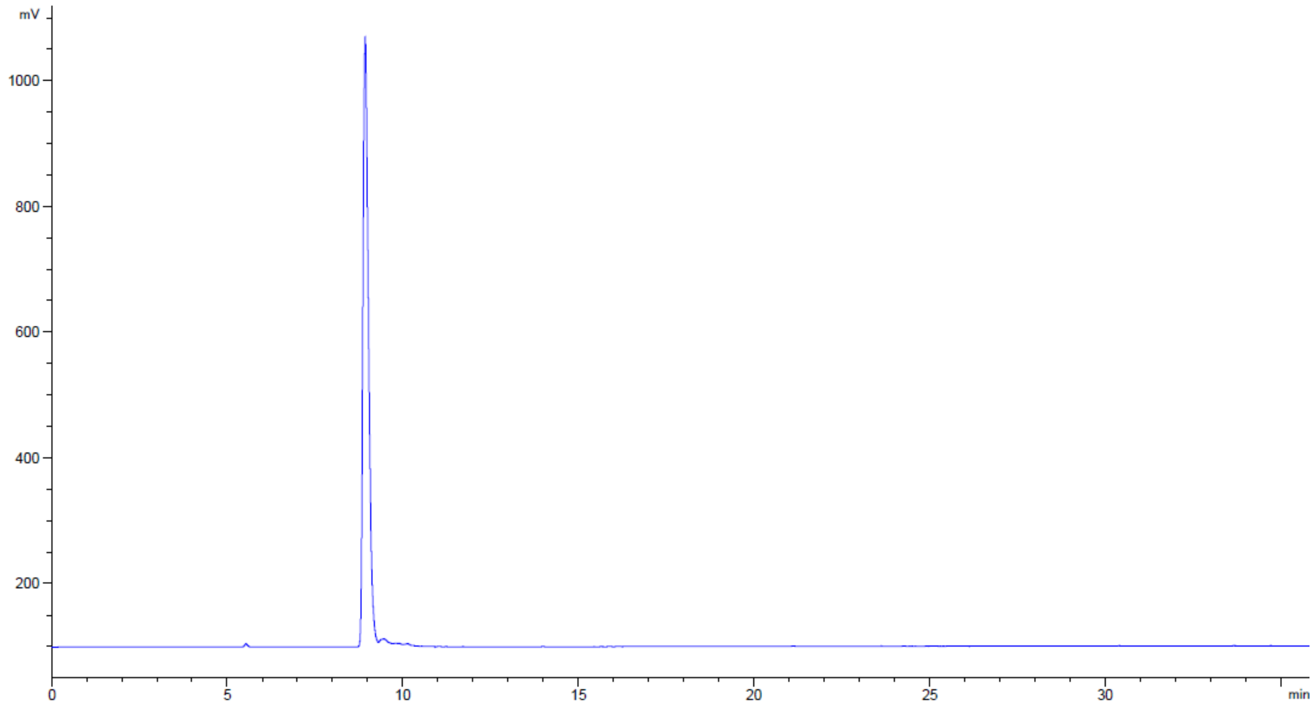

Synthesis of β-D-galactopyranosyl azide (4)

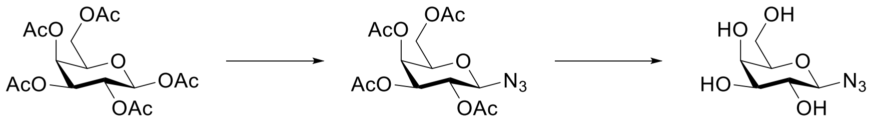

Molecule **4** was synthesized according to the references. (Pastuch-Gawołek et al., 2016; Tsoulougian et al., 2019)

2,3,4,6-Tetra-*O*-acetyl-β-D-galactopyranosyl azide (**13**) (450 mg, 2.71 mmol, 1.00 Equiv.) was dissolved in 8 mL of anhydrous methanol under argon atmosphere and 0.5 M sodium methoxide solution in methanol (0.80 mL) was added. The solution was stirred overnight at room temperature and then neutralized by Amberlite IR-120 H^+. The mixture was filtered over a pad of Celite and the solvent was removed under reduced pressure. The crude product was obtained as an off-white solid in 91% yield (225 mg, 1.09 mmol) and used without further purification.

^{1}H NMR (400 MHz, D_2O): δ = 3.46 (dd, *J* = 9.8, 8.6 Hz, 1H, *H*-2), 3.63 (dd, *J* = 9.9, 3.4 Hz, 1H, *H*-3), 3.69 – 3.77 (m, 3H, *H*-5, *H*-6α, *H*-6β), 3.91 (d, *J* = 3.4 Hz, 1H, *H*-4), 4.61 (d, *J* = 8.7 Hz, 1H, *H*-1)

ppm. **^{13}C NMR** (101 MHz, D_2O): δ = 60.8, 68.4, 70.2, 72.5, 77.1, 90.48 ppm. – HRMS (ESI): calcd for $C_6H_{11}N_3O_5$ Na $[M+Na]^+$: 228.0590; found: 228.0589.

The analytical data agree with the literature.(Pastuch-Gawołek et al., 2016; Tsoulougian et al., 2019)

RP-HPLC (ELSD trace, Method A, t_R= 3.9 min)

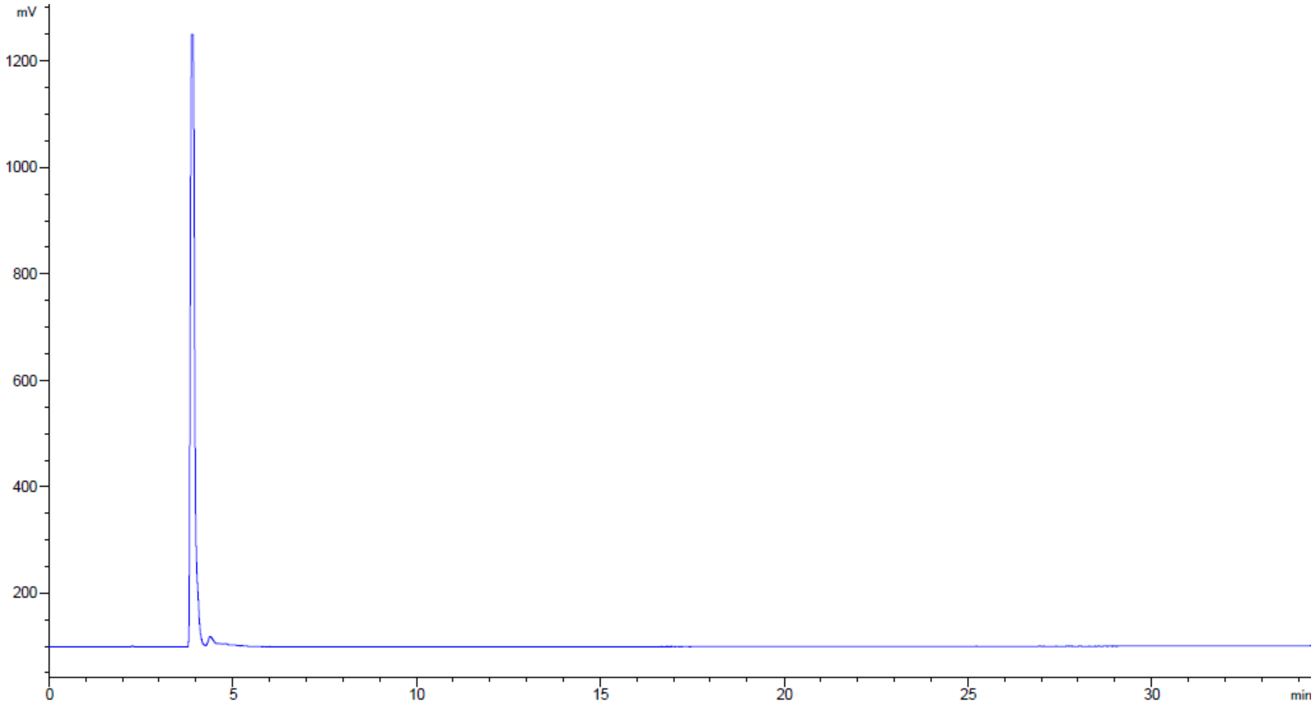

Synthesis of 2-(2-(2-azidoethoxy)ethoxy)ethyl-β-D-galactopyranoside (5)

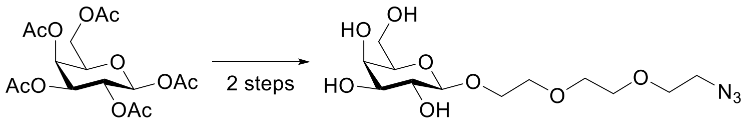

12

5

Molecule **5** was synthesized over 2 steps according to the reference. (Kong et al., 2015)

1(2-(2-(2-azidoethoxy)ethoxy)ethoxy)ethoxy 2,3,4,6-tetra-*O*-acetyl-β-D-galactopyranosyl azide (260 mg, 0.51 mmol, 1.00 Equiv.) was dissolved in 5 mL of anhydrous methanol and 0.5 M sodium methoxide solution in methanol (278 mg, 5.14 mmol) was added. The solution was stirred overnight

at room temperature and then neutralized by Amberlite IR-120 H^+. The mixture was filtered over a pad of Celite and the solvent was removed under reduced pressure. The crude product was obtained as a yellow oil in 97% yield (168 mg, 0.498 mmol).

^{1}H NMR (400 MHz, D_2O): δ = 3.46 (m, 3H, -CH_2N_3, *H*-2), 3.59 (d, *J* = 3.5 Hz, 1H, *H*-3), 3.60 – 3.81 (m, 12H,-OCH_2-, *H*-6α, *H*-5), 3.86 (d, *J* = 3.5 Hz, 1H, *H*-4), 4.00 – 4.06 (m, 1H, *H*-6β), 4.37 (d, *J* = 7.8 Hz, 1H, *H*-1) ppm. **^{13}C NMR** (101 MHz, D_2O): δ = 50.0, 60.8, 68.5, 69.1, 69.3, 69.9, 69.5, 70.6, 72.8, 75.7, 102.4 ppm. – **HRMS** (ESI): calcd for $C_{12}H_{23}N_3O_8$ Na $[M+Na]^+$: 360.1377; found: 360.1377.

The analytical data agree with the literature.(Kong et al., 2015)

RP-HPLC (ELSD trace, Method B, t_R= 12.8 min)

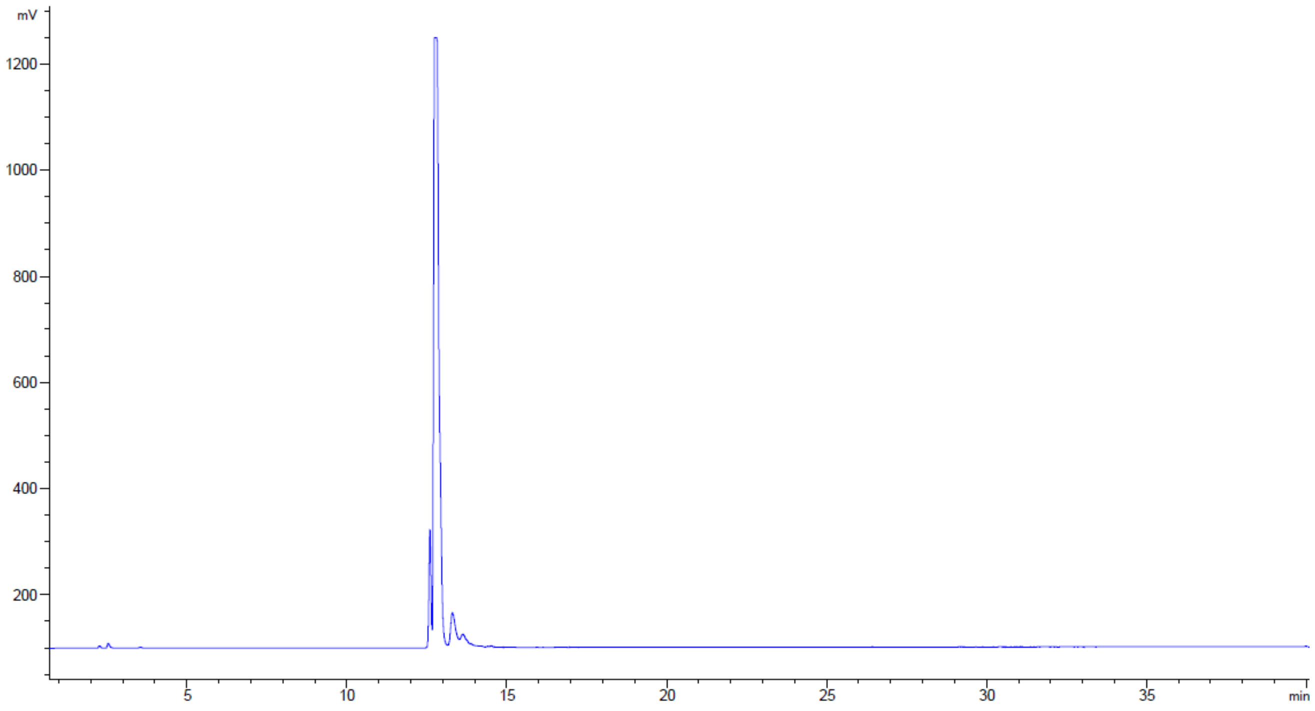

Synthesis of *N*-acetyl-β-D-galactosamine azide (6)

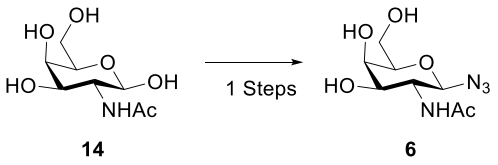

Molecule **6** was synthesized according to the reference.(Terada et al., 2019)

N-acetyl-β-D-galactosamine (50.00 mg, 0.23 mmol, 1.00 Equiv.) was dissolved in 1 mL of deuterium oxide. Sodium azide (146.92 mg, 2.26 mmol, 10.00 Equiv.) and 2.6-lutidine (0.24 mL, 2.03 mmol, 9.00 Equiv.) were added and the mixture was cooled down to 0°C. Then, 2-chloro-1,3-dimethylimidazolinium chloride (114.62 mg, 0.68 mmol, 3.00 Equiv.) was added and stirred for 1h in an ice bath. Progress of the reaction was confirmed by mass spectrometry. Water was removed under reduced pressure, ethanol was added and filtered off. Ethanol was removed under reduced pressure and the residue was dissolved in water. The solution was washed with dichloromethane five times, and the water was removed again under reduced pressure. The crude product was purified by reverse phase flash chromatography over a C_{18} cartridge with water and the product was obtained as a white solid in 90% yield (50 mg, 0.20 mmol)**.**

^{1}H NMR (400 MHz, MeOD): δ = 1.99 (s, 3H, Ac), 3.56 – 3.67 (m, 2H, *H*-3, *H*-5), 3.71 – 3.83 (m, 2H, *H*α-6, *H*β-6), 3.87 (dd, J = 3.2, 1.1 Hz, 1H, *H*-4), 3.93 – 4.02 (m, 1H, *H*-2), 4.44 (d, *J* = 9.3 Hz, 1H, *H*-1), 4.91 (brs, 3H, -OH) ppm. **^{13}C NMR** (101 MHz, MeOD): δ = 22.6, 53.1, 62.2, 69.3, 72.6, 78.8, 90.3, 173.8 ppm. – HRMS (ESI): calcd for $C_8H_{14}N_4O_5$ Na $[M+Na]^+$: 269.0856; found: 269.0859.

The analytical data agree with the literature.(Terada et al., 2019)

RP-HPLC (ELSD trace, Method C, t_R= 6.8 min)

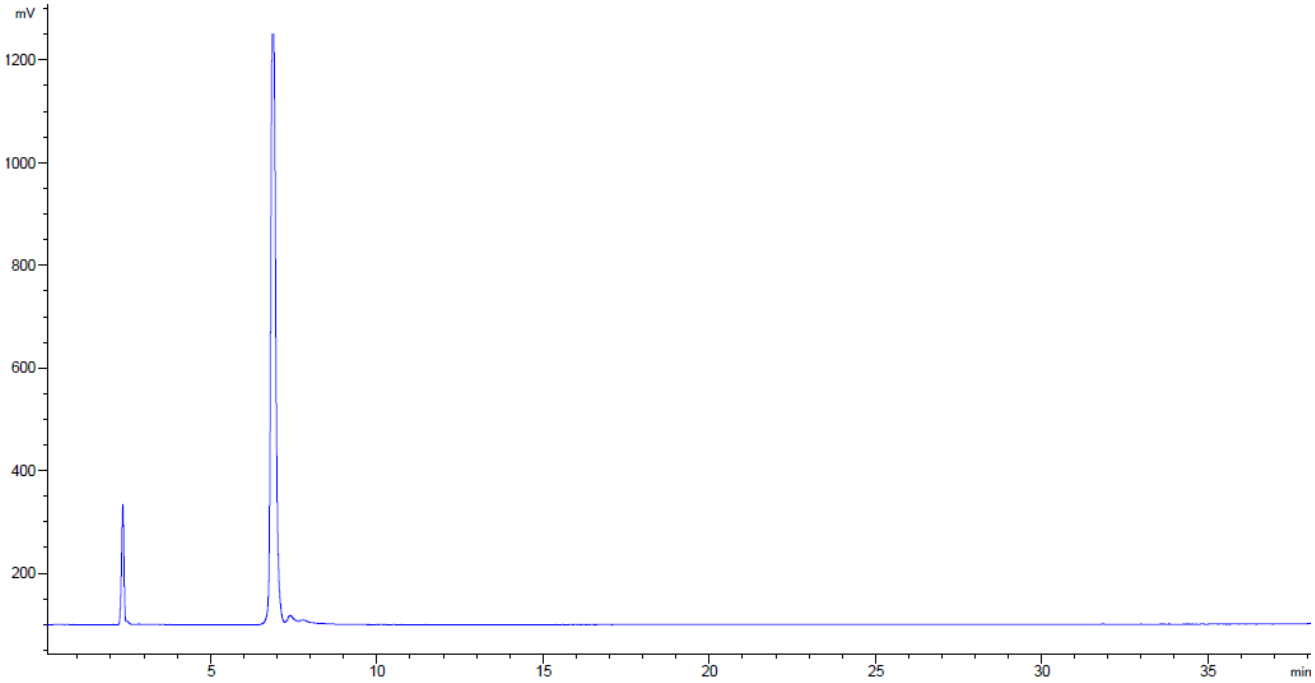

Synthesis of *N*-acetyl-β-D-glucosamine azide (8)

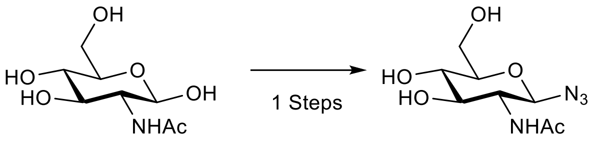

Molecule **8** was synthesized according to the reference.(Terada et al., 2019)

N-acetyl-β-D-glucosamine (100 mg, 0.45 mmol, 1.00 Equiv.) was dissolved in 2 mL of deuterium oxide. Sodium azide (293.88 mg, 4.52 mmol, 10.00 Equiv.) and 2.6-lutidine (0.47 mL, 4.01 mmol, 9.00 Equiv.) were added and the mixture was cooled down to 0°C. Then, 2-chloro-1,3-dimethylimidazolinium chloride (229.23 mg, 1.36 mmol, 3.00 Equiv.) was added and stirred for 1h in an ice bath. Progress of the reaction was confirmed by NMR and MS. Water was removed under reduced pressure, ethanol was added and filtered off. Ethanol was removed under reduced pressure and the residue was dissolved in water. The solution was washed with dichloromethane five times and the water was removed again under reduced pressure. The crude product was purified by reverse phase flash chromatography over a C_{18} cartridge with water and the product was obtained as a white solid in 70% yield (80 mg, 0.32 mmol).

^{1}H NMR (400 MHz, D_2O): δ = 1.91 (s, 3H, Ac), 3.29 – 3.36 (m, 1H, *H*-4), 3.37 – 3.41(m, 1H, H-5), 3.44 (dd, *J* = 8.3, 1.6 Hz, 1H, *H*-3), 3.55 (d, *J* = 9.6 Hz, 1H, *H*-2), 3.58 – 3.65 (m, 1H, *H*-6α), 3.78 (dd, *J* = 12.5, 2.2 Hz, 1H, *H*-6β), 4.61 (d, *J* = 9.2 Hz, 1H, *H*-1), 7.88 (d, *J* = 14.8 Hz, 1H, -N*H*Ac) ppm. ^{13}C NMR (101 MHz, D_2O): δ = 21.96, 54.89, 60.39, 69.33, 77.77, 88.60, 148.46 ppm. – HRMS (ESI): calcd for $C_8H_{14}N_4O_5$ Na $[M+Na]^+$: 269.0856; found: 269.0854.

The analytical data agree with the literature.(Terada et al., 2019)

RP-HPLC (ELSD trace, Method C, t_R= 9.5 min)

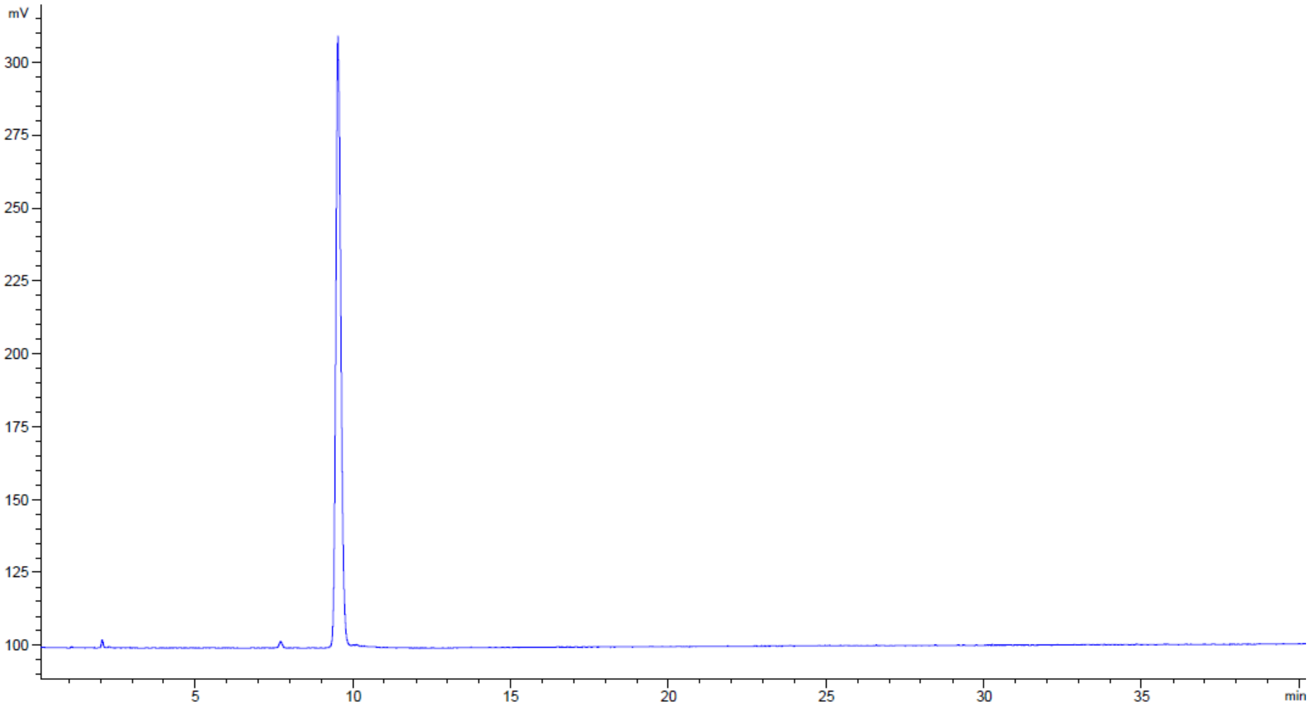

3 Laser transfer method

3.1 Donor slide preparation

Pre-activated amino acid (AA) Fmoc-glycine pentafluorophenyl ester, Fmoc-Gly-OPfp **1**, was used without prior activation, while the non-pre-activated amino acid Fmoc-L-2-propargylglycine, Fmoc-propargyl Gly-OH **16**, was prepared *in-situ* during the preparation of the spin-coating solution to form

the pentafluorophenyl (OPfp)-activated amino acid **2**. Pentafluorophenyl (OPfp)-activated amino acid was used without isolation or further characterization.

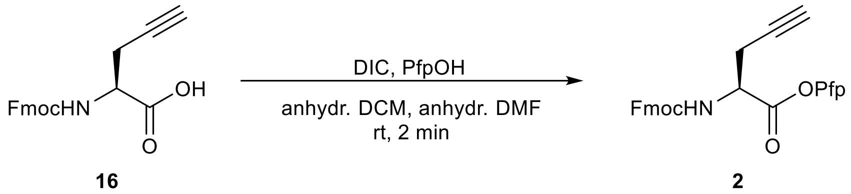

Fmoc-propargyl-Gly-OH (**16**) (3.00 mg, 9.00 µmol, 1.00 Equiv), *N,N'*-Diisopropylcarbodiimide (DIC) (1.4 µL, 9.00 µmol, 1.00 Equiv), Pentafluorophenol (PfpOH) (1.6 mg, 9.00 µmol, 1.00 Equiv) were dissolved in 50 µL anhydr. DMF in a vial, while 27 mg of inert polymer matrix (SLEC) were dissolved in 450 µL anhydr. DCM in another vial. The first solution containing the freshly activated amino acid was added into the second matrix solution. The final mixture was shaken for 2 min (vibrating orbital shaker) and afterwards the solution was spin-coated on top of the polyimide foil of the microscope glass slide, forming the thin layer of the transfer material.

3.2 Acceptor slide preparation

The commercially available slides used for our approach, 3D Fmoc-NH-β-Ala-PEGMA-co-MMA glass slide, PEPperSlide (**17)** from PEPperPRINT and 3D-Amino glass slides from PolyAn (**18**) are shown as simplified representations in the Figure below. *Fmoc-deprotection* of PEPperPRINT slides was needed to obtain the free amino groups on the glass slide functionalization.

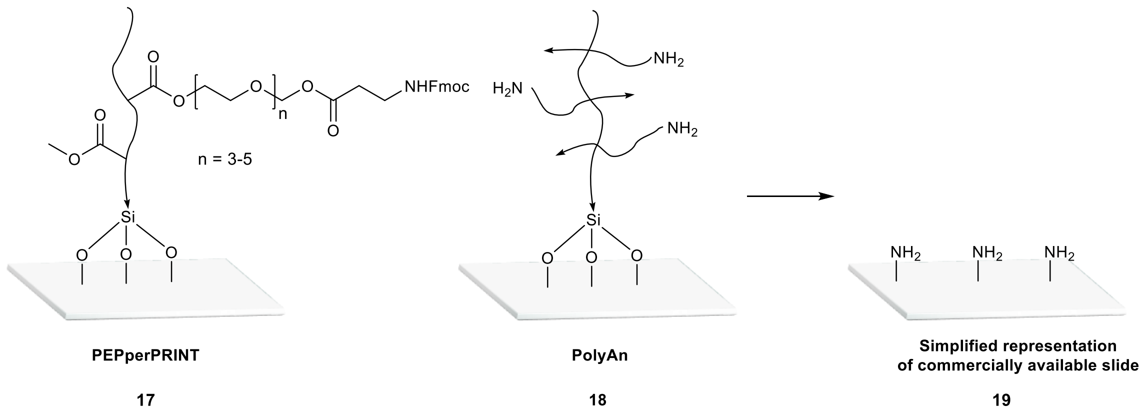

<u>*Fmoc-deprotection of PEPperPRINT slides:*</u>

The 3D Fmoc-protected glass slide **17** was pre-swelled in DMF for 20 min on a shaker in a petri dish. Afterwards, the slide was immersed in 10 mL of Fmoc-deprotection solution for 20 min on a shaker. The slide was washed and dried in a jet of air to obtain the free amino groups on the glass slide **19**.

3.2.1 Acceptor slides without spacer

After pre-swelling, slides without spacer were subjected to pre-patterning. Initially, laser transfer and coupling of two layers of glycine was performed. Afterwards, the synthesis of the desired tetrapeptide scaffolds followed.

3.2.2 Acceptor slides with spacer

<u>Spacer attachment:</u>

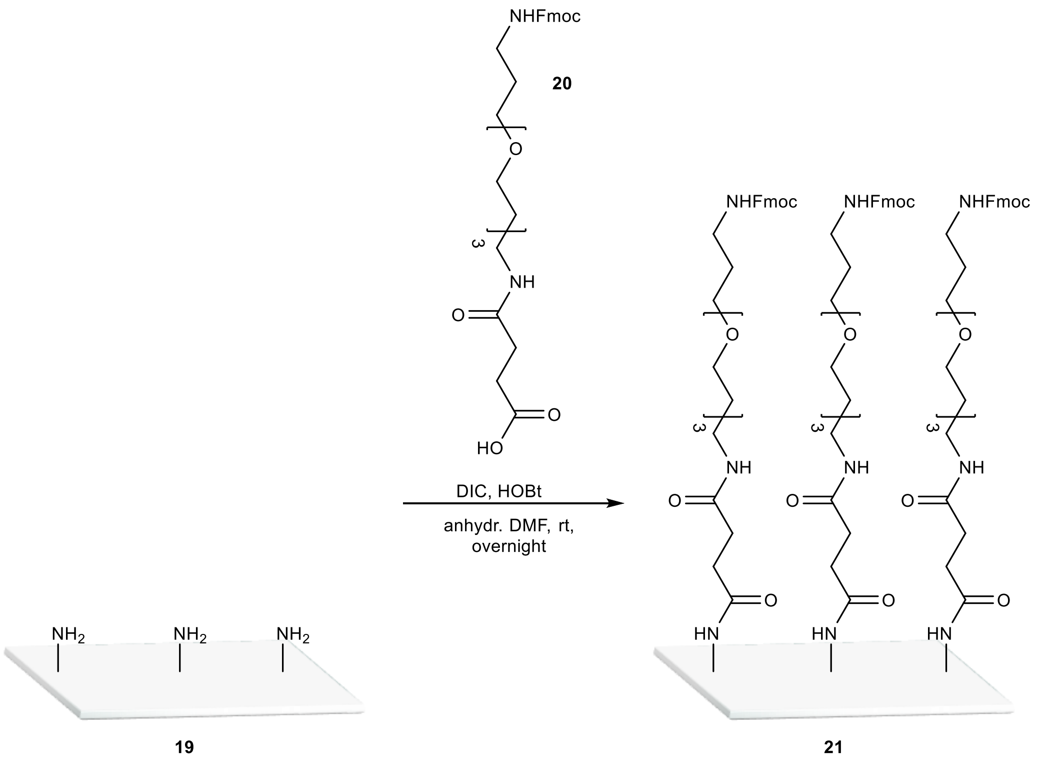

N-Fmoc-*N″*-succinyl-4,7,10-trioxa-1,13-tridecanediamine (**20**) (Fmoc-TTDS-OH) (27.0 mg, 50 µmol, 1.00 Equiv.) was dissolved in 250 µL of anhydrous DMF (peptide grade) in a vial. *N,N'*-diisopropylcarbodiimide (DIC) (23.2 µL, 18.9 mg, 150 µmol, 3.00 Equiv.) and hydroxybenzotriazole (HOBt) (6.76 mg, 50 µmol, 1.00 Equiv.) were added consecutively and the vial was shaken for a few seconds. The resulting solution was pipetted on the free amino glass slide **19** and another slide was

placed on top of the first one (sandwich functionalization method). The slides were left overnight in a petri dish to react, and then washed and dried in a jet of air to obtain spacer-functionalized glass slide **21**.

Capping of unreacted amine groups:

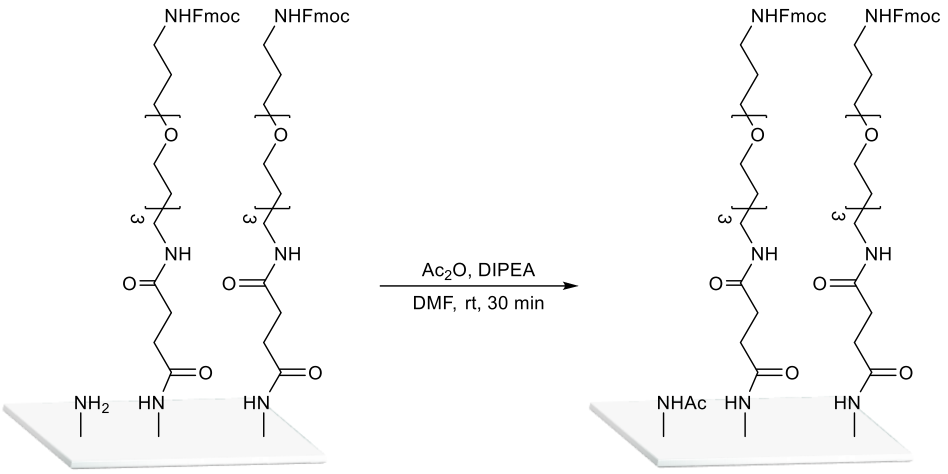

21 **22**

The remaining unreacted free NH_2 groups on the acceptor slide were subjected to acetylation for 30 min. The same process was repeated with a freshly prepared capping solution for another 30 min at room temperature (300 rpm). Then, the slide was dried in a jet of air to obtain the Fmoc-protected amine-spacer-functionalized glass slides **22** with capped unreacted amine groups.

Fmoc-deprotection:

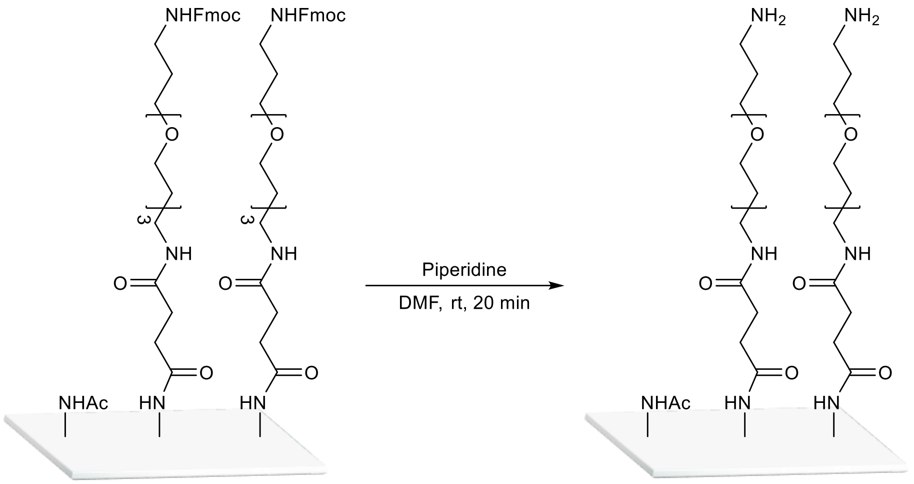

22 **23**

Deprotection of the Fmoc-spacer-functionalized glass slides was achieved after treatment of the slide with the deprotection solution for 20 min (shaking 300 rpm). Afterwards, the glass slide was washed and dried in a jet of air to obtain the desired amine-spacer-functionalized glass slide **23**.

4 Parameter optimization for the synthesis of tetrapeptides

For a successful synthesis of tetrapeptides on the different solid supports, several parameters had to be optimized. The key points that had to be investigated were: (a) the amino acid concentration in the donor slide (b); the laser power and pulse duration during the laser transfer of the material onto the solid support; (c) the coupling time inside the oven to optimize the coupling efficiency.

4.1 PEPperPRINT slide optimization

In a previous study,(Mende et al., 2020) the synthesis of tetrapeptides was performed predominantly on PEPperPRINT slides with the same amino acids but different donor slide concentrations. For the generation of the donor slides bearing the already activated Fmoc-Gly-OPfp **1**, 3.00 mg of the L-amino acid building block and 27.0 mg of inert polymer matrix was used. In case of the non-activated Fmoc-Pra-OH (**16**), *in-situ* activation was achieved by using 2.00 mg of L-amino acid and 18.0 mg of inert polymer matrix. Moreover, we found that our synthesis quality increased if the lasing parameters were set to 80 mW lasing power and 6 ms pulse duration, while the coupling should be performed for 60 min at 95 °C. Recently, it was even observed that the coupling time could be reduced from 60 min to 10 min for all amino acids (data not shown, data are part of a different publication). To ensure a homogeneous spot size throughout the synthesis, we initially compared the coupling efficiency of both amino acids for 10 min and 60 min coupling time. We performed a single amino acid transfer of a (lasing power/pulse duration (x/y)) gradient pattern on PEPperPRINT slides, coupling at 95 °C for 10 min and 60 min. Then, staining was carried out of the transferred glycine (**1**) and propargylglycine (**2**) with DyLight™ 633-B2 NHS ester and TAMRA azide dye **24** respectively. Spot size increase was observed for a coupling time of 10 min compared to the 60 min. However, screening of the stained propargylglycine structures was challenging, since no fluorescence signal could observed under these conditions. Self-quenching of the dye due to the energy transfer mechanism is one of the main reported challenges.(Shiba et al., 2017) To reduce the quenching effect, the PEPperPRINT slide was functionalized with a PEG-based spacer and pre-patterned with two layers of glycine prior to propargylglycine coupling. Again, transfer of the propargylglycine was performed using 80 mW and

6 ms pulse duration. Staining of propargylglycine showed that the obtained spot size of the pre-patterned glycine is larger than the size of propargylglycine spots. This can also be visually detected by the formation of a light green shade around each spot (**Supplementary Figure 1**). These shades appear to become smaller at 60 min oven coupling time compared to 10 min. To avoid visible shades around the spots for our peptide synthesis, equal amounts of Fmoc-Gly-OPfp (**1**) and non-activate Fmoc-Pra-OH (**16**) (3 mg of amino acid and 27 mg of inert matrix) were chosen and the coupling step of each layer was repeated twice to increase the synthetic yield and maintain the quality of the synthesized structures.

**Amino acid**	Fmoc-Gly-OPfp **1**		Fmoc-Pra-OPfp **2**	
**Laser parameters**	Gradient pattern<br>Power: 45 - 77.5 %, 60 - 100mW<br>ΔP: 2.5 %<br>values: 14<br>Dot time: 1 ms<br>Δt: 0.3 ms<br>values: 31	Gradient pattern<br>Power: 45 - 77.5 %, 60 - 100mW<br>ΔP: 2.5 %<br>values: 14<br>Dot time: 1 ms<br>Δt: 0.3 ms<br>values: 31	Power: 60 %, 80 mW<br>6 ms pulse duration	
**Pitch [μm]**	250	250	250	250
**Oven coupling [min]**	10	60	10	60
**Surface modification**	No modification	No modification	PEG-based spacer-Gly-Gly	PEG-based spacer-Gly-Gly
**Spot size [cm²]**	1.77×10^{-4}	1.20×10^{-4}	5.6×10^{-5}	4.3×10^{-5}

A **B** **C** **D**

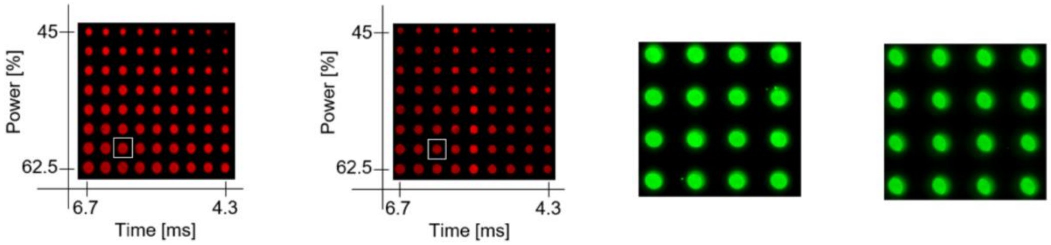

Supplementary Figure 1: Comparison between stained glycine and propargylglycine spots on PEPperPRINT surfaces. (A) Single transfer of a glycine gradient pattern and 10 min coupling (laser power/pulse duration) on a PEPperPRINT slide (spot at 6 ms and 60 % power marked with a white square). (B) Single transfer of a glycine gradient pattern and 60 min coupling (laser power/pulse duration) on a PEPperPRINT slide (spot at 6 ms and 60 % power marked with a white square). Only an extract of the gradient pattern is shown. (A, B) (C) Single transfer of Fmoc-Pra-OPfp **2** on a pre-modified PEPperPRINT slide, and 10 min coupling time. (D) Single transfer of Fmoc-Pra-OPfp **2** on a pre-modified PEPperPRINT slide, and 60 min coupling time. Scanning parameters: Wavelengths 635/532 nm, PMT gain 400/600, power 33 %, pixel size 5 µm. Distance between centers of two spots (pitch) is 250 µm.

4.2 PolyAn slide optimization

We only synthesized peptides on PEPperPRINT slides and there is no reported data for the peptide synthesis on PolyAn slides. Therfore, different parameter combinations had to be investigated. For the preparation of the donor slides, the previously used amounts for the amino acids and inert matrix (3 mg of AA and 27 mg of inert polymer matrix) were used. Again, a gradient pattern was transferred from the AA donor slides onto unmodified and PEG-spacer modified PolyAn slides. In this case, we tested 10 min and 30 min coupling time (data not shown, data are part of a different publication). No quenching effects were observed for the TAMRA azide dye labeling of propargylglycine. Furthermore, no difference in spot size was recognizable, with or without spacer modification of the surface with both coupling times. However, the laser power, as used on PEPperPRINT surfaces (80 mW), lead to blurry and overlapping of spots. Thus, an adjusted gradient transfer was performed with a subsequent coupling time of10 min to reduce overall array manufacturing time and maintain the spot size. To investigate the number of required coupling repetitions within one amino acid layer for an optimal synthesis, the transfer of the gradient pattern and consecutive coupling was carried out three times on a Polyan slide bearing a PEG-based spacer (**Supplementary Figure 2)**. To achieve comparable spot sizes between glycine and propargylglycine on PolyAn slides, lasing parameters of 60 mW and 6 ms were used. Three transfers seemed to be sufficient of one layer for good overall coupling quality. Moreover, the spot sizes of both amino acids after one transfer are comparable to the spot sizes of glycine on PEPperPRINT slides.

**Amino acid**	Fmoc-Gly-OPfp **1**	Fmoc-Pra-OPfp **2**
**Laser parameters**	Gradient pattern<br>Power: 40 – 48 %, 50 - 62 mW	Gradient pattern<br>Power: 40 – 48 %, 50 - 62 mW

	ΔP: 1 %<br>values: 9<br>Dot time: 1 ms<br>Δt: 0.5 ms<br>values: 9			ΔP: 1 %<br>values: 9<br>Dot time: 1 ms<br>Δt: 0.5 ms<br>values: 9		
**Pitch [µm]**	250			250		
**Oven coupling [min]**	10			10		
**Surface modification**	PEG-based spacer			PEG-based spacer		
**No. of transfers**	I	II	III	I	II	I
**Spot size [cm^2]**	1.84×10^{-4}	2.28×10^{-4}	3.18×10^{-4}	1.17×10^{-5}	2.28×10^{-5}	2.22×10^{-5}
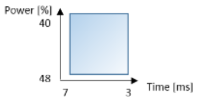	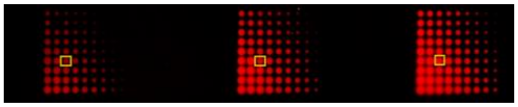			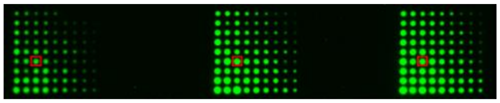		

Supplementary Figure 2: Comparison between glycine and propargylglycine spots on PolyAn surfaces. Up to three coupling repetitions of a gradient transfer of both amino acids is shown. Scanning parameters: Wavelengths 635/532 nm, PMT gain 400/600, power 33%, pixel size 5 µm. Distance between centers of two spots (pitch) is 250 µm for each microarray..

To determine the spot size of propargylglycine in a similar manner as on PEPperPRINT slides, a PolyAn slide was modified with a spacer and two layers of glycine. This was followed by a transfer of propargylglycine using 60 mW of laser power and a pulse duration of 6 ms. A spot size of 1.45×10^{-4} [cm^2] was calculated which is comparable in size with the results on PEPperPRINT slides (**Supplementary Figure 3**).

**Amino acid**	Fmoc-Pra-OPfp **2**
**Laser parameters**	P: 46 %, 60 mW, 6 ms pulse duration
**Oven coupling [min]**	10

**Surface modification**	PEG-based spacer-Gly-Gly	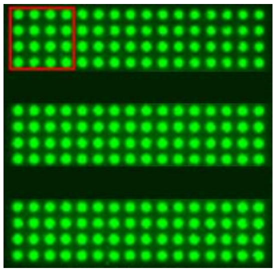
**No. of transfers**	III	Spot size: 1.45x10^{-4}[cm^2]

Supplementary Figure 3: Spot size of coupled propargylglycine on PolyAn surfaces bearing the same modification as the PEPperPRINT slides (PEG-spacer-Gly-Gly). Transfer and coupling of the same pattern was repeated three times, using the respective lasing conditions. Scanning parameters: Wavelength 532 nm, PMT gain 400, laser power 33 %, pixel size 5 µm. Distance between centers of two spots (pitch) is 250 µm.

5 Quality control of synthesized microarrays

The quality of the synthesized microarrays was tested by attaching a TAMRA azide dye **24** to the peptide backbone to compare the intensities of each spot.(Mende et al., 2020) On the PEPperPRINT slides with PEG-spacer, a rather constant fluorescence intensity was observed. In our previously reported data, sequences with more than one propargylglycine showed less intensity due to self-quenching of multiple adjacent dye molecules, based on the FRET mechanism.(Shiba et al., 2017) This trend was this time much less observed on the PEPperPRINT slides. The newly optimized conditions for the preparation of the donor slides and the reduced oven coupling time seem to give better microarray quality (due to generally stronger binding of lectins), but interestingly with less quenching (details will be published elsewhere). The further the Pra is away (peptide sequence wise, C to N terminus) from the solid support, the higher fluorescence intensity was obtained. Similar results were observed for the two PolyAn slides with and without PEG-spacer. On the PolyAn slide with spacer, higher staining intensities were observed. This might be due to a larger distance between solid support and clicked dye molecules, making the peptide scaffold more accessible for the dye. Additionally, a higher quenching is observed on PolyAn slides with spacer than on PolyAn slides without spacer. Lower intensities were detected with more molecules of propargylglycine attached on PolyAn slides with PEG-spacer. On the PolyAn slides without spacer, a linear increase on the binding was detected, based on the position of Pra on the monovalent scaffolds. In comparison to PEPperPRINT surfaces, both PolyAn slides show higher intensities, **Supplementary Figure 4**.

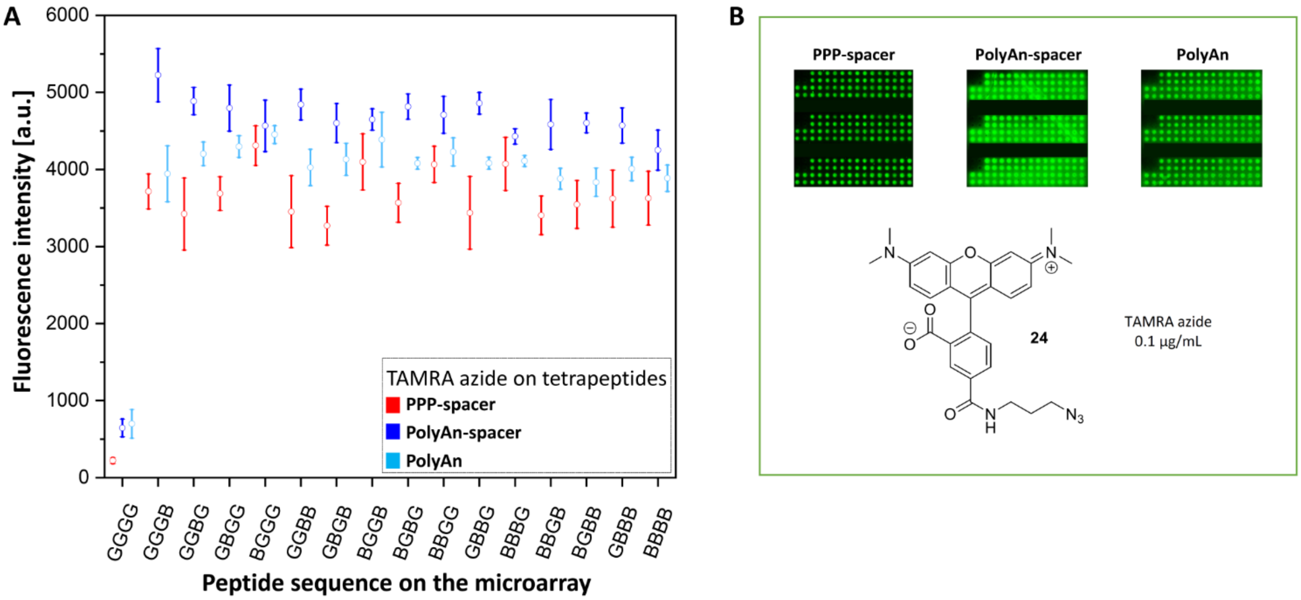

Supplementary Figure 4: (A) Fluorescence staining intensities, (B) fluorescence scan images of the different tetrapeptides with TAMRA azide dye **24** on our different surfaces. Scanning parameters: Wavelength 532 nm, PMT gain 400, laser power 33 %, pixel size 5µm. Spots pitch is 250 µm.

CuAAC of TAMRA azide dye 24 on the peptide backbone: $CuSO_4$ (530 µg, 3.36 µmol, 2.00 equiv.) and sodium ascorbate (998 µg, 5.04 µmol, 3.00 equiv.) were added to a mixture of 100 µL DMSO and 100 µL water in a vial. The vial was shaken for 5 min and, afterwards, the precipitate was centrifuged and the remaining solution was passed through a syringe filter (0.2 µm, polypropylene, diameter 25 mm, Whatman). TAMRA azide was dissolved in this solution (concentration of 0.1 µg/mL) and, then, applied on the acceptor surface (c = 8.4 µmol/mL). For the incubation, a 16-well format incubation chamber (PEPperPRINT GmbH, Heidelberg/Baden-Württemberg, Germany) was used. The resulting solution (200 µL) was pipetted in one of the wells and the microarray was incubated overnight in the dark at rt (150 rpm). Afterwards, the slide was washed with water inside the chamber (3 × 5 min), in a petri dish (1 × 30 min), and dried in a jet of air.

6 Contact angle measurements

The contact angle, and as an extent, the hydrophilicity of the substrates, was measured with the setup described in the following reference (Karpitschka et al., 2017). Two droplets of water (15 µL and 50 µL) were deposited on each substrate, and the contact angle of the drops was derived from simultaneous imaging from the side and the top, between the sample and the drop. The top view shows the circularity of the drop and the overall motion of the droplet during measurement. The cross section, and the distortion-free depiction of each droplet was derived from the telecentric view. The contact

angle was determined and fitted with a home built spherical cap. The gravity in all these experiments was neglected, since each droplet size was smaller than the total length of the capillary used. The typical error during the measurements was estimated to ~0.1°. For the entire experiment, the time between the droplet deposition and the measurement was minimized to avoid evaporation effects (< 20s). From our experiments, we observed that the PolyAn slides are more hydrophilic than the PEPperPRINT slides. Additionally, between the PolyAn slides with spacer and without spacer, there is no significant difference on the hydrophobicity of the surface. However, the attachment of the spacer has a significant effect on the hydrophilicity of the substrate on PEPperPRINT slides, since it decreases the hydrophilicity of the surface, **Supplementary Figure 5**.

A. PEPperPRINT

A.1. PEPperPRINT without functionalization

H_2O Droplet: 15 µL 50 µL

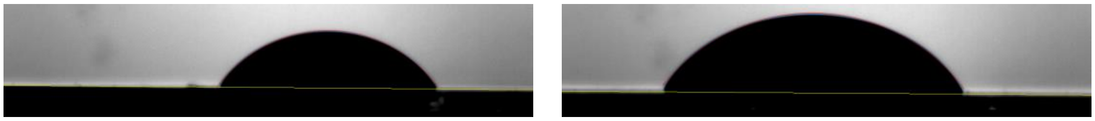

Angle [°]: $\theta = 54.5 \pm 0.0°$ $\theta = 54.6 \pm 0.0°$

A.2. Functionalized PEPperPRINT with PEG-spacer (PPP-spacer)

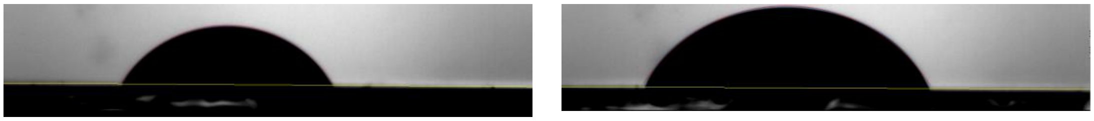

Angle [°]: $\theta = 57.6 \pm 0.0\,°$ $\theta = 58.5 \pm 0.0\,°$

***B.* PolyAn**

B.1. PolyAn without functionalization

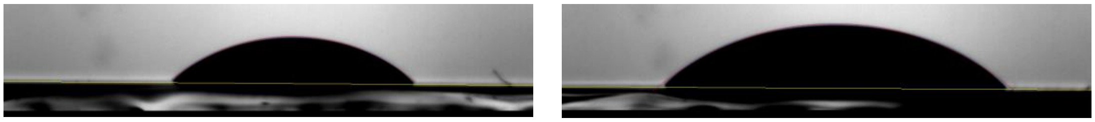

Angle [°]: $\theta = 41.0 \pm 0.1°$ $\theta = 39.8 \pm 0.1°$

B.1. Functionalized PolyAn with spacer (PolyAn-spacer)

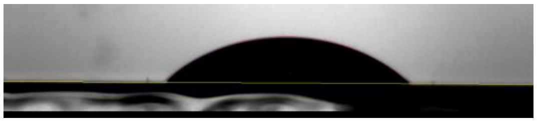

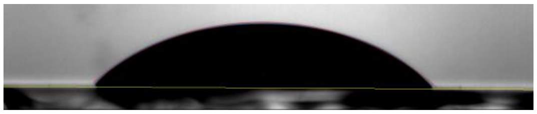

Angle [°]: $\theta = 40.4 \pm 0.1°$ $\theta = 40.9 \pm 0.1°$

Supplementary Figure 5: Side view image of water droplets on different functionalized and non-functionalized PEPperPRINT and PolyAn slides. (A.1) PEPperPRINT slide without functionalization, (A.2.) functionalized PEPPERPRINT slide with PEG-spacer, (B.1.) PolyAn slide without functionbalization and (B.2.) functionalized PolyAn slide with PEG-spacer.

7 Binding assays of plant lectins

7.1 Concanavalin A (ConA)

In this work, due to optimized synthetic conditions, the staining intensities are one order of magnitude higher from our previously published results.(Mende et al., 2020) In this previous work, the donor slides were prepared using 2 mg of amino acid and 18 mg of polymer matrix whereas here with 3 mg of amino acid and 27 mg of matrix, and the oven coupling time was reduced from originally 60 min to 10 min, indicating that the longer coupling time might have led to damage of the peptide backbone or to loss of reactivity due to extended heat exposure (95 °C). Evaluation of the fluorescence intensities of PolyAn slides with PEG-spacer functionalization showed around 50% less binding than the PEPperPRINT slides with the same functionalization. This observation might be explained by the reduced laser power used for the transfer of the amino acids (25 % less power on PolyAn). This might have potentially resulted in a lower amount of amino acid transfer, leading to a smaller number of available peptide structures to click the sugar azides. Consequently, fewer ConA lectins bound on the glycopeptide array. In addition, a higher fluorescence intensity was found on the non-functionalized PolyAn surface compared to the PolyAn slide with the PEG-spacer. In general, PolyAn surfaces are known to be less protein resistant in comparison to the PEPperPRINT surfaces(Stadler et al., 2008) due to their more hydrophilic functionalization (see also Section 6). On the PolyAn slides with PEG-spacer, the hydrophilicity was higher than on the PolyAn slides without a spacer. Instead, it was observed that the prior functionalization has no significant effect on the hydrophilicity of the modified- and non-modified array of PolyAn. Legume lectin binding depends on hydrophobic interactions,(Loris

et al., 1998) which might explain the lower binding intensity on the PolyAn-spacer slides in comparison to the PolyAn without functionalization, leading to diminished binding of ConA. The binding profile of ConA was the same on all slide functionalizations.

7.2 Peanut agglutinin (PNA) binding assay

For the PNA binding assay, β-Gal azide **4** and the β-Gal-PEG3 azide **5** were used. At a concentration of 10 μg/mL, no binding of PNA on the PEPperPRINT slide with spacer was observed, whereas on PolyAn surfaces with and without spacer, a really weak interaction was seen. To have a better understanding of this weak interaction, the staining was repeated with higher concentration of lectin, 100 μg/mL. Similarly, on the PEPperPRINT surface, no binding was visible, and weak fluorescence intensities were observed for both PolyAn slides. The observed hydrophobic interactions are present between the lectin and the peptide backbone. The structure of this non-glycosylated lectin has been extensively studied. The exact distance between the carbohydrate recognition domains (CRDs) and the recognition mechanism has been also investigated. (Ravishankar et al., 1997; Natchiar et al., 2007) In case of the β-Gal azide **4**, the azide in the anomeric position might disturb the formation of all seven hydrogen bonds needed for successful recognition of the sugar moiety, while the hydrophobicity of the PEPperPRINT surface could diminish the lectin binding. Moreover, no binding was detected for β-Gal-PEG3 azide **5**, although the PEG3 spacer should give better accessibility and flexibility to the sugar. We believe that the distances between the sugar and the peptide backbone, as well the length of backbone are too small/short for our monosaccharides to bind in a cross-linking multivalent mode. **Supplementary Figure 6** summarizes the results.

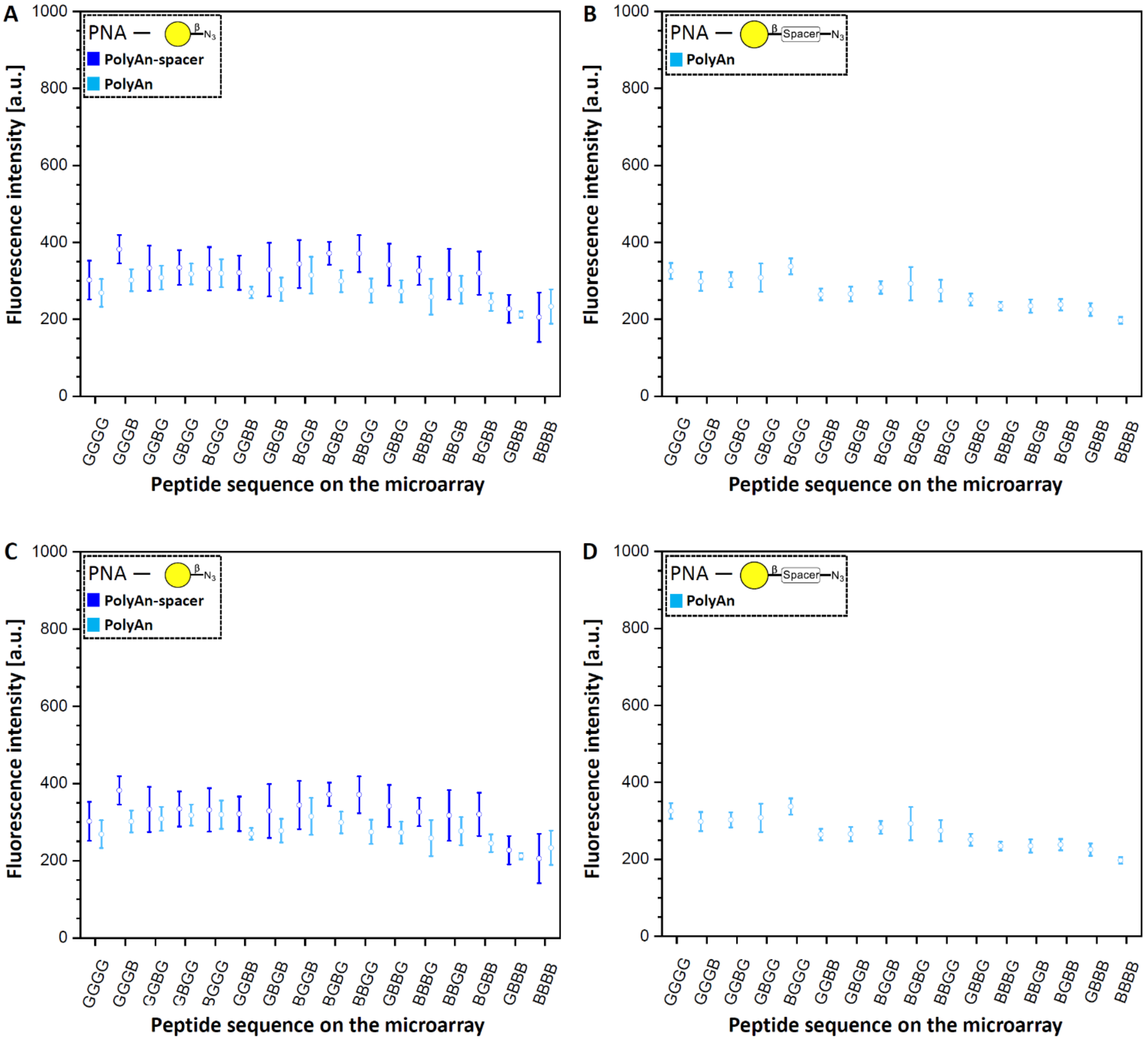

Supplementary Figure 6: Fluorescence staining intensities of respective sugar azides **4** and **5** with PNA (rhodamine labeled, 10 µg/mL and 100 µg/mL lectin concentration): (A) β-galactose azide **4** with 10 µg/mL lectin concentration, (B) β-galactose PEG3-spacer azide **5** with 10 µg/mL lectin concentration, (C) β-galactose azide **4** with 100 µg/mL lectin concentration, and (D) β-galactose PEG3-spacer azide **5** with 100 µg/mL lectin concentration, on PEPperPRINT slides with PEG-spacer (PPP-spacer), and on PolyAn functionalized slides with (PolyAn-spacer) and without PEG-spacer (PolyAn**)**.

7.3 Dolichos biflorus Agglutinin (DBA) binding assay

DBA is a glycoprotein with specificity towards terminal non-reducing N-acetyl galactosamine carbohydrates (GalNAc), showing unique high specificity toward α-GalNAc moieties. To test the binding of DBA in our tetrapeptide system, we used the β-GalNAc azide **6** and β-GalNAc-PEG3 azide **7**. The chosen lectin concentration for all our experiments was 10µg/mL. Our goal was to determine

whether binding on beta residues can be achieved via changes on the position of the anomeric center and on different environments (hydrophilic, hydrophobic substrates).(Hamelryck et al., 1999) On our PEPperPRINT slides, no interaction was detected, while on our PolyAn surfaces, we identified a weak interaction with the peptide backbone (GGGG) for both sugar azides. To better understand this interaction, we compared the staining obtained from the glycopeptides with DBA and the plain peptides with DBA. From our experiments, we noticed that the further apart the sugar moiety is from the peptide scaffold, the better is the binding between lectin and glycopeptides. The fluorescence was rather constant in the plain peptide and not as high as for the screened glycopeptides. Less binding is possible on our hydrophilic surfaces, resulting in weak lectin binding (**Supplementary Figure 7**). For a better understanding, in the future, screening of the α-GalNAc moieties on our surfaces should be performed.

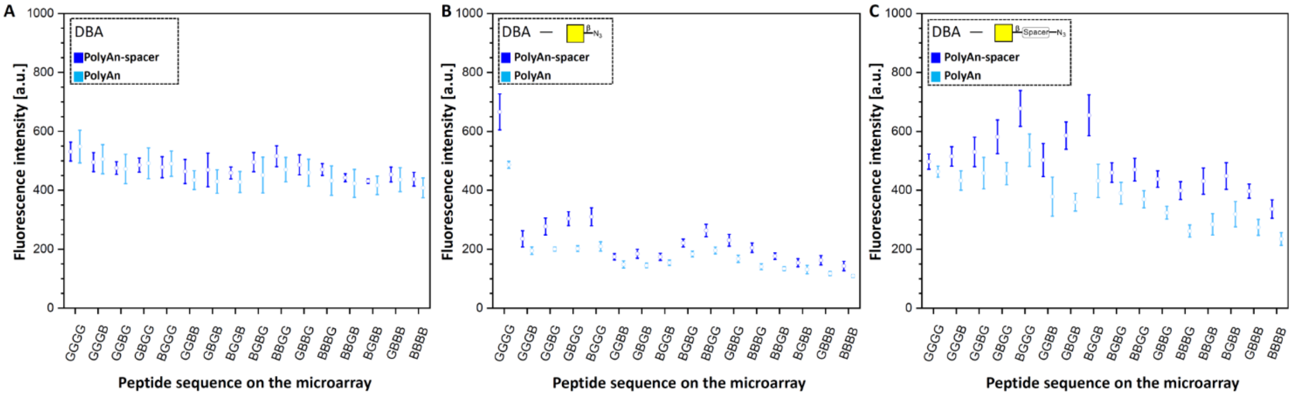

Supplementary Figure 7: Fluorescence staining intensities of (A) plain peptide scaffolds, (B) sugar β-GalNAc azide **6**, and (C) β-GalNAc-PEG3 azide **7**, with DBA (rhodamine labeled, 10 µg/mL concentration) on PolyAn functionalized slides with (PolyAn-spacer) and without (PolyAn) PEG-spacer.

7.4 Soybean agglutinin (SBA) binding assay

According to literature, SBA is a GalNAc-/Gal-specific tetrameric legume lectin with similar binding sites as PNA and DBA. For our studies, we used β-Gal azide **4**, β-Gal-PEG3 azide **5**, β-GalNAc azide **6**, and β-GalNAc-PEG3 azide **7**. In the case of sugar azides **4**, **5**, and **6**, no binding was identified on all surfaces (results not shown). As mentioned in literature, binding between the SBA tetramers and the terminal β-galactose involves cross-linking of the sugar to symmetry-related neighboring molecules, potentially explaining our unsuccessful staining. Notably, increased binding of GalNAc **7** on all used substrates was pointed out. This derives from the fact that the amino sugar participates in a

hydrogen bond network formation between the acetate group of the sugar and the side chain of the asparagine (Asp) 88 of the lectin. Another reason might be the increased distance given from the PEG-spacer, between the sugar moiety and the formed triazol ring during the CuAAC, minimizing hydrogen bonds that can occur between the triazol ring and the Asp 88 of the lectin.(Pereira et al., 1974; Dessen et al., 1995).

7.5 Ricinus communis agglutinin I (RCA-I) binding assay

To evaluate the binding of RCA-I on our tetrapeptide scaffolds, β-Gal **4** and β-Gal-PEG3 azide **5** were used. In general, the PEPperPRINT surface showed higher fluorescence intensity compared to both PolyAn surfaces, due to its hydrophobic nature. Comparing both PolyAn slides, stronger binding was seen on the more hydrophilic PolyAn slide without spacer. On the functionalized slides with the PEG-spacer, RCA-I showed similar binding trends on the simplest β-Gal azide **4**. The highest binding was achieved for the tetravalent sugar display. Contrarily, on the PolyAn surface without spacer, a linear increase in binding was detected. On the monovalent scaffold, the fluorescence intensity increased linearly with the position of the propargylglycine in the peptide tetramer. The further the propargylglycine was apart from the solid support, the more intense the fluorescence signal was (strongest binding for BGGG). A similar behavior was observed for the divalent and the trivalent scaffolds. Strongest binding on the divalent system was detected for the structures having two terminal sugar azides (BBGG), and three terminal sugar azides (BBBG) for the trivalent system, whereas less intensity was observed on the scaffolds with an intermediated glycine (BBGB, BGBB). Based on these findings, it is assumed that the binding mechanism between RCA-I and the β-Gal azide **4** relies on the statistical/proximity effect. Especially in the mono- and divalent structures, this binding type might be predominant, because of the easier accessibility of the sugars. The formation of hydrogen bonds between the hydroxyl groups and the CRD is facilitated due to unimpeded presentation of the sugars themselves. A chelating binding mechanism seems to be less likely because the propargylglycines in the peptide scaffold are at maximum 11 Å apart. The CRDs of RCA-I display a distance of ≈100 Å.(Wittmann and Pieters, 2013) The PEG3-spacer on the galactose azide **5** results in increased flexibility of the sugar moiety on the peptide scaffold, making the sugar more accessible. Increased fluorescence intensity was identified on the PEPperPRINT surface. Particularly on the monovalent scaffolds, the average increase of fluorescence was about 55%. The linear increase in the intensity observed for β-Gal **4** was not observed for β-Gal-PEG3 azide **5** on the PolyAn slides without the spacer. The flexible β-Gal-PEG3 azide **5** adopted a multivalent binding trend, and the binding intensity showed

exponential increase based on the number of valencies on the scaffold. This change in binding on the PolyAn slide without spacer could be explained by the increased accessibility and flexibility due to the PEG3-spacer within the sugar-azides. Thereby, RCA-I gains accessibility to the azide with the PEG-spacer.

7.6 Wheat germ agglutinin (WGA) binding assay

For the binding assay of WGA on our tetrapeptides, β-GlcNAc azide **8** and β-GlcNAc-PEG3 azide **9** were used. The initially used lectin concentration, 10 µg/mL, led to high saturation of WGA on our tri- and tetravalent tetrapeptide scaffolds on the hydrophilic PolyAn microarrays, whereas it was better controlled on the hydrophobic PEPperPRINT slides. In particular, on the PEPperPRINT surface, the spots of the formed glycopeptides were easier to observe than on the PolyAn slides. Despite this effect, the binding trend of WGA was the same on all used slides for the β-GlcNAc azide **8**. On the monovalent structures, increased fluorescence intensities were detected for scaffolds with terminal Pra (BGGG, GGGB). We observed that spacing plays an important role on the binding of WGA: in the divalent systems, scaffolds with two neighboring Pra amino acids (GGBB, BBGG, GBBG) showed less binding intensities, whereas structures with non-neighboring Pra amino acids (GBGB, BGGB, BGBG) show stronger binding. The theoretical spacing of the scaffolds with two adjacent Pra is calculated to be ≈ 2.1 nm, while in the non-adjacent ≈ 0.7 nm and ≈ 2.3 nm.(Mende et al., 2020) Binding on the trivalent system indicated that the intermediate Gly (BBGB, BGBB) increases the binding strength, in comparison to the structures with terminal glycine (GBBB, BBBG), **Supplementary Figure 8**. To avoid saturation, the concentration of WGA was decreased 50-fold (0.2 µg/mL). The intensities of the stained glycopeptides were 2.5-fold lower at 0.2 µg/mL compared to 10 µg/mL WGA, with no significant change on the recognition pattern trend. For the monovalent scaffolds, linear increase of fluorescence was obtained with the position of the Pra in the peptide tetramer (GGGB > GGBG > GBGG > BGGG), which is inverse to most other lectins. On the bivalent system, the same binding mode was detected, with the highest intensity for the divalent largest spacing system of Pra (BGGB). The divalent scaffolds with the same spacing gave the same result (GBGB, BGBG). Similarly, on the tri- and tetravalent systems, the same binding pattern was attained as explained previously. Thus, it can be concluded that we have a surface and concentration dependent binding for WGA on the tetrapeptides to β-GlcNAc azide **8** due to the chelating effect (**Figure 3E** & **F**). The spacing of the sugars may fit well to the spacing of the CDRs.

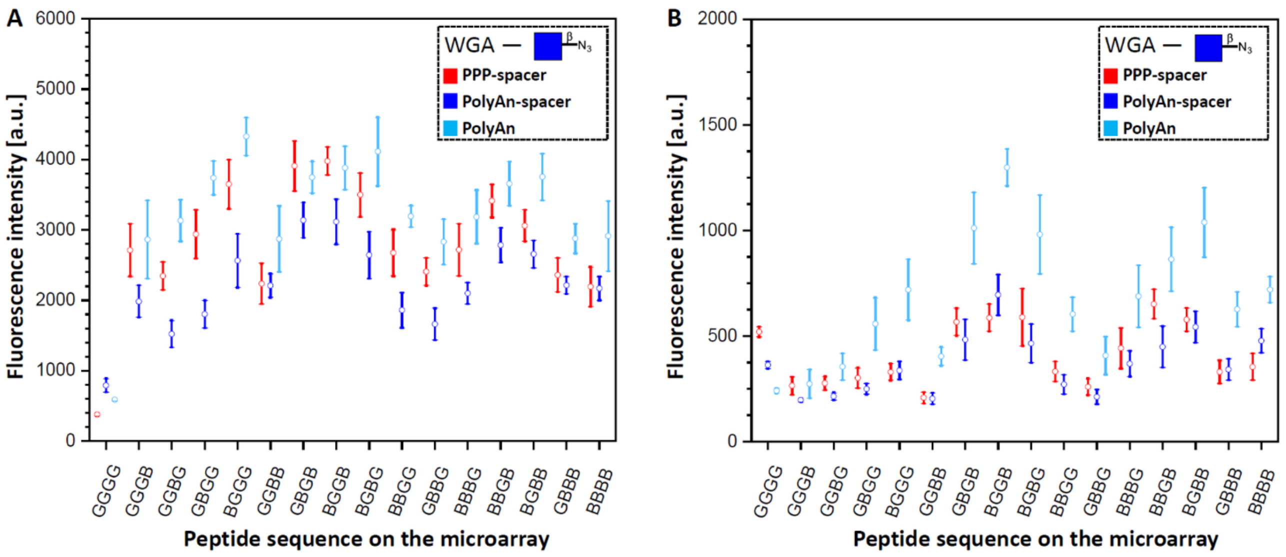

Supplementary Figure 8: Fluorescence staining intensities of β-GlcNAc azide with WGA: (A) β-GlcNAc azide **8** (10 µg/mL lectin concentration), (B) β-GlcNAc azide **8** (0.2 µg/mL lectin concentration) on PEPperPRINT functionalized slide with PEG-spacer (PPP-spacer|) and on PolyAn slide with (PolyAn-spacer) and without (PolyAn) PEG-spacer respectively.

8 Binding assay of C-type lectins

For the binding assay of the mouse-derived C-type lectins (mLangerin, mMincle, and mMGL-1), we used our sugar monomers: α-Man **3**, β-Gal **4**, β-GalNAc **6**, β-GlcNAc **8**, and our synthesized α- and β-Glc (Mende et al., 2020) azides, as well as the more flexible β-Gal-PEG3 azide **5**. Langerin is a type II C-type lectin expressed from Langerhans cells with specificity towards mannose, fucose and GlcNAc moieties. Similarly, the macrophage inducible Ca^{2+}-dependent lectin receptor (Mincle) binds a range of carbohydrate moieties containing Glc or Man, while the macrophage galactose-type lectin (MGL) shows specificity towards Gal and terminal GalNAc moieties.(Artigas et al., 2017; Mayer et al., 2017; Valverde et al., 2020) All used lectins were fused with human Fc antibody fragment(Maglinao et al., 2014).

Structures containing the α-Man azide **3** were incubated with mLangerin and mMincle. Glycopeptides with β-Gal **4**, β-Gal-PEG3 azide **5**, and β-GalNAc **6** were incubated with mMGL-1, and structures containing α- and β- Glc incubated with mMincle. Then, slides were washed with the staining buffer for 1 min. Secondary antibody staining to screen the result was performed using goat polyclonal anti-human IgG Fc DyLight™ 650. Unfortunately, despite screening multiple lectin concentrations, buffers, and using different concentrations of anti-human IgG (1:1000 and 1:500), it was not possible for us to detect any binding. We believe that due to the low density (Dam and Brewer, 2010) of the multivalent

glycopeptides on the solid support, no binding could be observed. In addition, the low valency of our structures might be one of the reasons for insufficient binding. In the future, sugar dimers, such as Manα1.2Man, should be used for mLangerin and mMincle, as used by Di Maio *et al.*.(Di Maio et al., 2021) Direct labeling of the C-type lectins might also give a better result, as well the use of hLangerin as in our previously reported work with highly specific binders.(Mende et al., 2020) In the following table, we summarize all applied conditions and results (**Supplementary Table 1**).

Supplementary Table 1: Glycan-Glycan binding protein assays with C-type lectins. No binding is represented (-).

**Entry**	**Lectin**	**Conc.** **[**μg/mL]	**Specificity** [term. sugar]	**Buffer**	**Results**
**1**	mLangerin	10	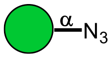	50 mM HEPES, 100 mM NaCl, 1 mM $CaCl_2$, 1 mM $MnCl_2$, 10% blocking buffer, 0.05% Tween 20, pH 7.5	×
**2**	mLangerin	10	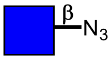	50 mM HEPES, 100 mM NaCl, 1 mM $CaCl_2$, 1 mM $MnCl_2$, 10% blocking buffer, 0.05% Tween 20, pH 7.5	×
**3**	mMincle	10	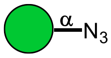	50 mM HEPES, 100 mM NaCl, 1 mM $CaCl_2$, 1 mM $MnCl_2$, 10% blocking buffer, 0.05% Tween 20, pH 7.5	×
**4**	mMincle	10	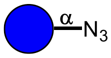	50 mM HEPES, 100 mM NaCl, 1 mM $CaCl_2$, 1 mM $MnCl_2$, 10% blocking buffer, 0.05% Tween 20, pH 7.5	×
**5**	mMincle	10	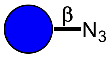	50 mM HEPES, 100 mM NaCl, 1 mM $CaCl_2$, 1 mM $MnCl_2$, 10% blocking buffer, 0.05% Tween 20, pH 7.5	×

**6**	mMGL-1	10	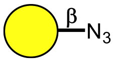	50 mM HEPES, 100 mM NaCl, 1 mM $CaCl_2$, 1 mM $MnCl_2$, 10% blocking buffer, 0.05% Tween 20, pH 7.5	×
**7**	mMGL-1	10	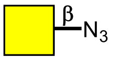	50 mM HEPES, 100 mM NaCl, 1 mM $CaCl_2$, 1 mM $MnCl_2$, 10% blocking buffer, 0.05% Tween 20, pH 7.5	×
**8**	mLangerin	30	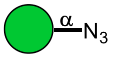	50 mM HEPES, 100 mM NaCl, 2 mM $MnCl_2$, 5 mM $CaCl_2$, 1 mM $MgCl_2$, 10% blocking buffer, 0.05% Tween 20, pH 7.5	×
**9**	mLangerin	60	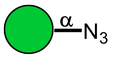	10 mM HEPES, 1 mM $MgCl_2$, 1 mM $CaCl_2$, 0.01% Tween 20, 2% BSA, pH 7.42	×
**10**	mMGL-1	10	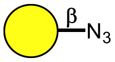	10 mM HEPES, 1 mM $MgCl_2$, 1 mM $CaCl_2$, 0.01 % Tween 20, 2% BSA, pH 7.42	×
**11**	mMGL-1	10	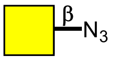	10 mM HEPES, 1 mM $MgCl_2$, 1 mM $CaCl_2$, 0.01% Tween 20, 2% BSA, pH 7.42	×
**12**	mMGL-1	10	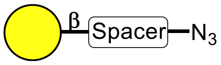	10 mM HEPES, 1 mM $MgCl_2$, 1 mM $CaCl_2$, 0.01% Tween 20, 2% BSA, pH 7.42	×
**13**	mMGL-1	60	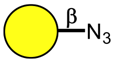	10 mM HEPES, 1 mM $MgCl_2$, 1 mM $CaCl_2$, 0.01% Tween 20, 2% BSA, pH 7.42	×
**11**	mMGL-1	60	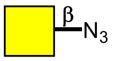	10 mM HEPES, 1 mM $MgCl_2$, 1 mM $CaCl_2$, 0.01% Tween 20, 2% BSA, pH 7.42	×

9 Fluorescence scan images

In the following **Supplementary Figures 9-28**, the fluorescence scan images of all synthesized and screened glycopeptides are shown after incubation of each structure with the corresponding

fluorescently labeled lectin (ConA, RCA-I, PNA, SBA, DBA, and WGA). The plain peptides were used as negative controls.

Concanavalin A (ConA)

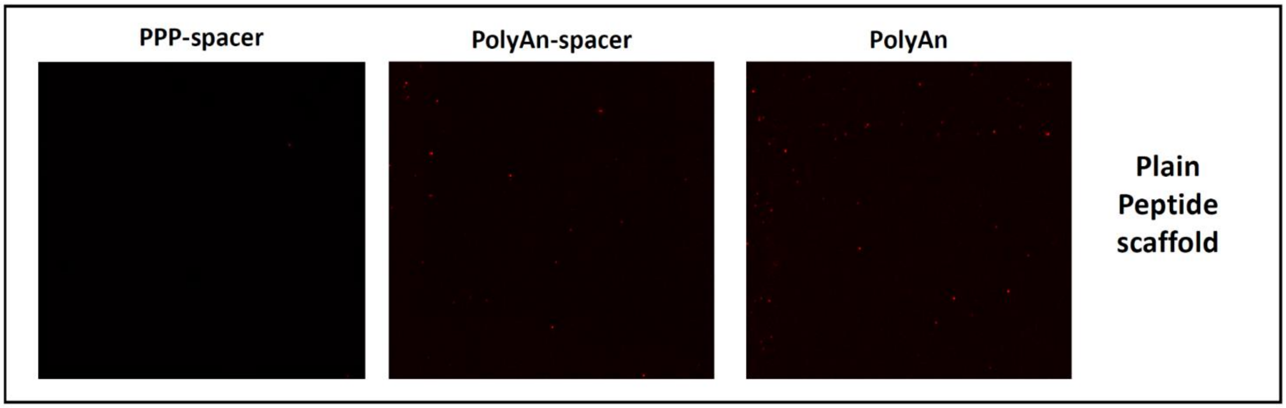

Supplementary Figure 9: Fluorescence scan image of the plain peptides (negative control) after incubation with CF®633 ConA. Incubation was performed in HEPES-buffer (50 mM HEPES, 100 mM NaCl, 1 mM $CaCl_2$, 1 mM $MnCl_2$, 10 % blocking buffer and 0.05 % Tween-20, pH = 7.5) at 100 µg/mL ConA concentration. Scanning parameters: Wavelength 635 nm, PMT gain 600, laser power 33 %, pixel size 5 µm. Spot pitch is 250 µm.

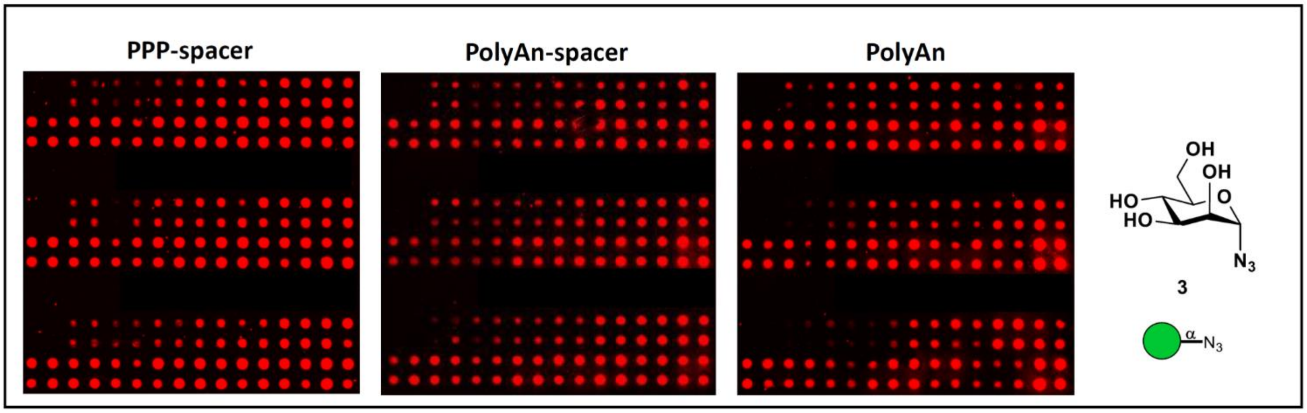

Supplementary Figure 10*:* Fluorescence scan image of glycopeptides containing Man azide **3** after incubation with CF®633 ConA. Staining was performed in HEPES-buffer (50 mM HEPES, 100 mM NaCl, 1 mM $CaCl_2$, 1 mM $MnCl_2$, 10 % blocking buffer and 0.05 % Tween-20, pH = 7.5) at 100 µg/mL ConA concentration. Scanning parameters: Wavelength 635 nm, PMT gain 600, laser power 33 %, pixel size 5 µm. Spot pitch is 250 µm.

Ricinus Communis Agglutinin I (RCA-I)

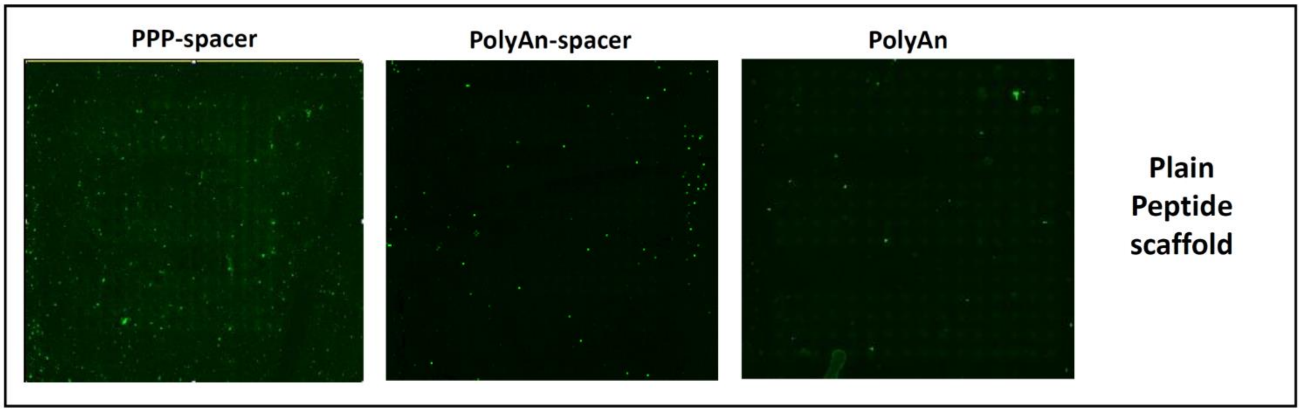

Supplementary Figure 11: Fluorescence scan image of the plain peptides (negative control) after incubation with Rhodamine-labelled RCA-I. Incubation was performed in HEPES-buffer (50 mM HEPES, 100 mM NaCl, 1 mM $CaCl_2$, 1 mM $MnCl_2$, 10 % blocking buffer and 0.05 % Tween-20, pH=7.5) at 10 µg/mL lectin concentration. Scanning parameters: Wavelength 532 nm, PMT gain 500, laser power 33 %, pixel size 5 µm. Spot pitch is 250 µm.

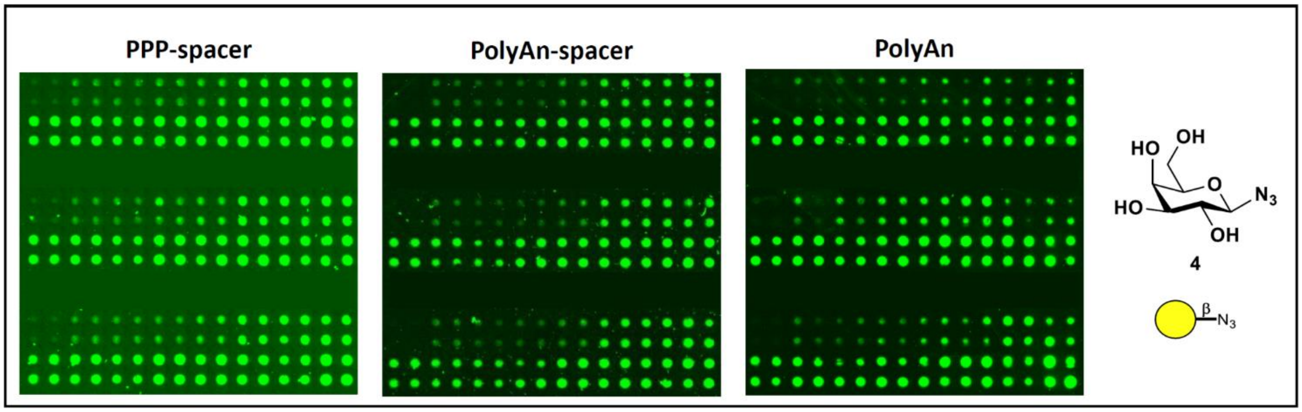

Supplementary Figure 12: Fluorescence scan image of glycopeptides containing β-Gal azide **4** after incubation with Rhodamine-labelled RCA-I. Staining was performed in HEPES-buffer (50 mM HEPES, 100 mM NaCl, 1 mM $CaCl_2$, 1 mM $MnCl_2$, 10 % blocking buffer and 0.05 % Tween-20, pH = 7.5) at 10 µg/mL RCA-I concentration. Scanning parameters: Wavelength 532 nm, PMT gain 500, laser power 33 %, pixel size 5 µm. Spot pitch is 250 µm.

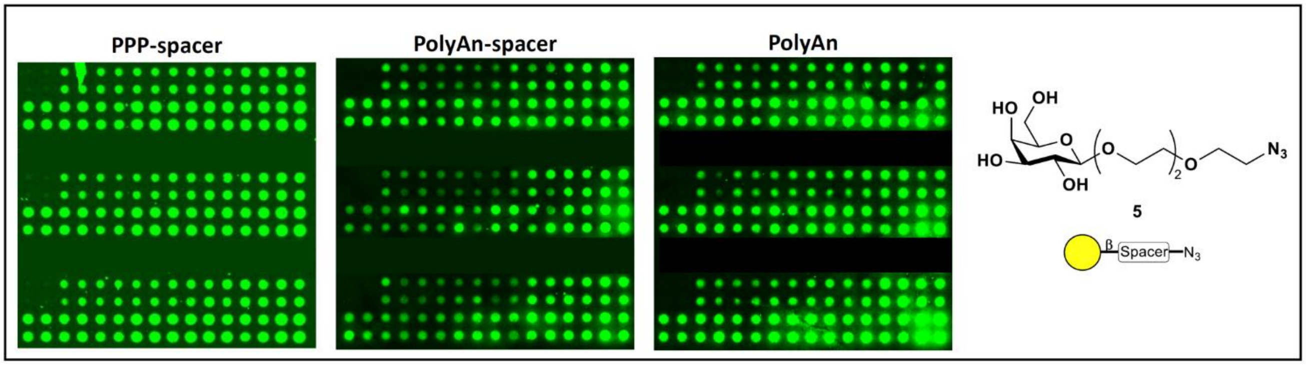

Supplementary Figure 13: Fluorescence scan image of glycopeptides containing β-Gal-PEG3 azide **5** after incubation with Rhodamine-labelled RCA-I. Staining was performed in HEPES-buffer (50 mM HEPES, 100 mM NaCl, 1 mM $CaCl_2$, 1 mM $MnCl_2$, 10 % blocking buffer and 0.05 % Tween-

20, pH = 7.5) at 10 µg/mL RCA-I concentration. Scanning parameters: Wavelength 532 nm, PMT gain 500, laser power 33 %, pixel size 5 µm. Spot pitch is 250 µm.

Peanut Agglutinin (PNA)

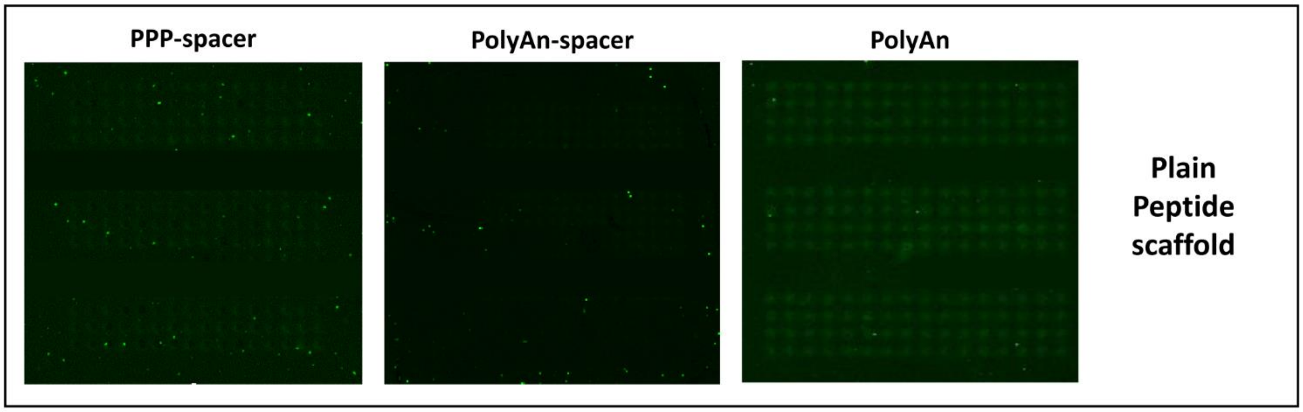

Supplementary Figure 14: Fluorescence scan image of the plain peptides (negative control) after incubation with Rhodamine-labelled PNA. Incubation was performed in HEPES-buffer (50 mM HEPES, 100 mM NaCl, 1 mM $CaCl_2$, 1 mM $MnCl_2$, 10 % blocking buffer and 0.05 % Tween-20, pH = 7.5) at 10 µg/mL lectin concentration. Scanning parameters: Wavelength 532 nm, PMT gain 500, laser power 33 %, pixel size 5 µm. Spot pitch is 250 µm.

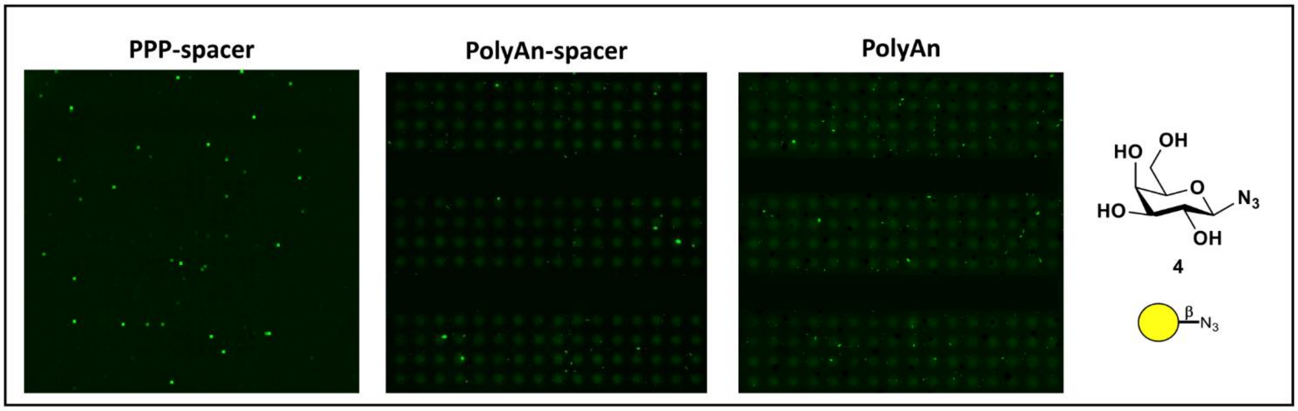

Supplementary Figure 15: Fluorescence scan image of glycopeptides containing β-Gal azide **4** after incubation with Rhodamine-labelled PNA. Staining was performed in HEPES-buffer (50 mM HEPES, 100 mM NaCl, 1 mM $CaCl_2$, 1 mM $MnCl_2$, 10 % blocking buffer and 0.05 % Tween-20,

pH = 7.5) at 100 µg/mL PNA concentration. Scanning parameters: Wavelength 532 nm, PMT gain 500, laser power 33 %, pixel size 5 µm. Spot pitch is 250 µm.

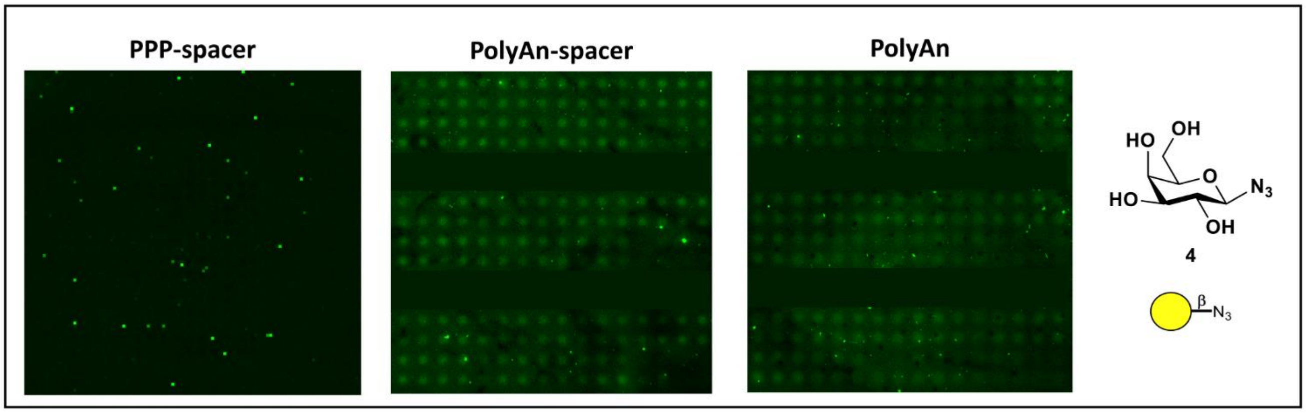

Supplementary Figure 16: Fluorescence scan image of glycopeptides containing galactose **4** after incubation with Rhodamine-labelled PNA. Staining was performed in HEPES-buffer (50 mM HEPES, 100 mM NaCl, 1 mM $CaCl_2$, 1 mM $MnCl_2$, 10 % blocking buffer and 0.05 % Tween-20, pH = 7.5) at 10 µg/mL PNA concentration. Scanning parameters: Wavelength 532 nm, PMT gain 500, laser power 33 %, pixel size 5 µm. Spot pitch is 250 µm.

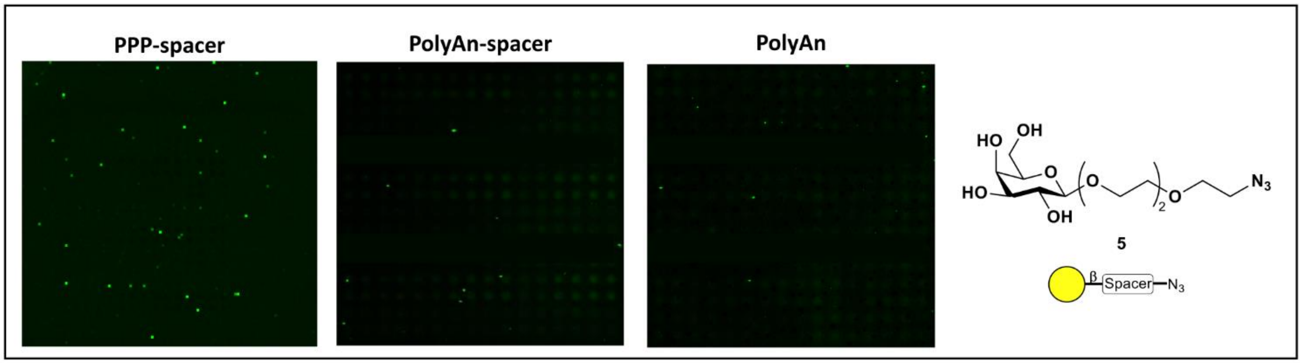

Supplementary Figure 17: Fluorescence scan image of glycopeptides containing β-Gal-PEG3 azide **5** after incubation with Rhodamine-labelled PNA. Staining was performed in HEPES-buffer (50 mM HEPES, 100 mM NaCl, 1 mM $CaCl_2$, 1 mM $MnCl_2$, 10 % blocking buffer and 0.05 % Tween-20, pH = 7.5) at 100 µg/mL PNA concentration. Scanning parameters: Wavelength 532 nm, PMT gain 500, laser power 33 %, pixel size 5 µm. Spot pitch is 250 µm.

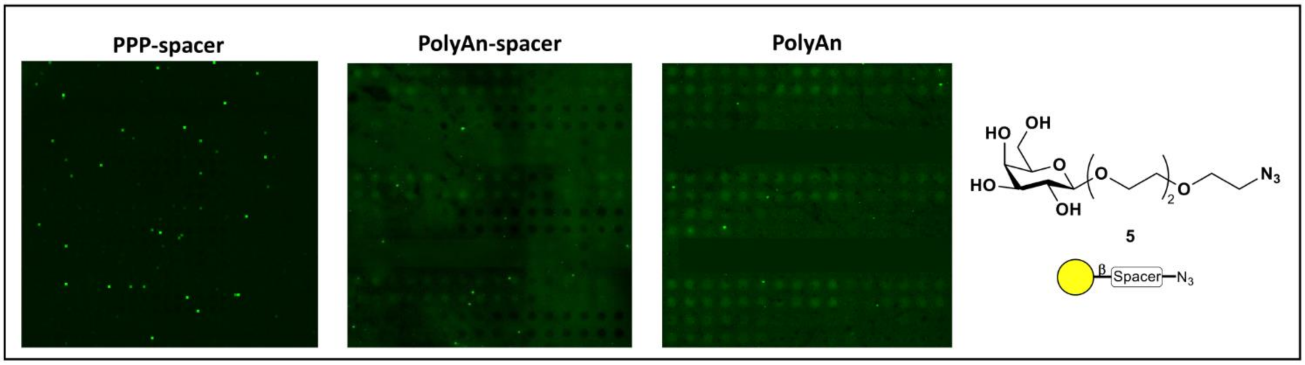

Supplementary Figure 18: Fluorescence scan image of glycopeptides containing β-Gal-PEG3 azide **5** after incubation with Rhodamine-labelled PNA. Staining was performed in HEPES-buffer (50 mM

HEPES, 100 mM NaCl, 1 mM $CaCl_2$, 1 mM $MnCl_2$, 10 % blocking buffer and 0.05 % Tween-20, pH = 7.5) at 10 µg/mL PNA concentration. Scanning parameters: Wavelength 532 nm, PMT gain 500, laser power 33 %, pixel size 5 µm. Spot pitch is 250 µm.

Soybean Agglutinin (SBA)

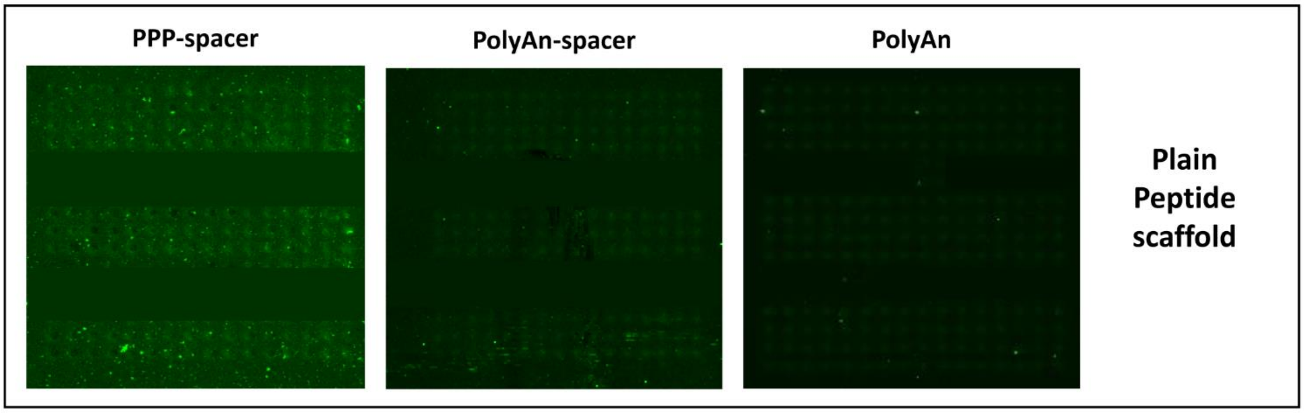

Supplementary Figure 19: Fluorescence scan image of the plain peptides (negative control) after incubation with Rhodamine-labelled SBA. Incubation was performed in HEPES-buffer (50 mM HEPES, 100 mM NaCl, 1 mM $CaCl_2$, 1 mM $MnCl_2$, 10 % blocking buffer and 0.05 % Tween-20, pH = 7.5) at 10 µg/mL lectin concentration. Scanning parameters: Wavelength 532 nm, PMT gain 500, laser power 33 %, pixel size 5 µm. Spot pitch is 250 µm.

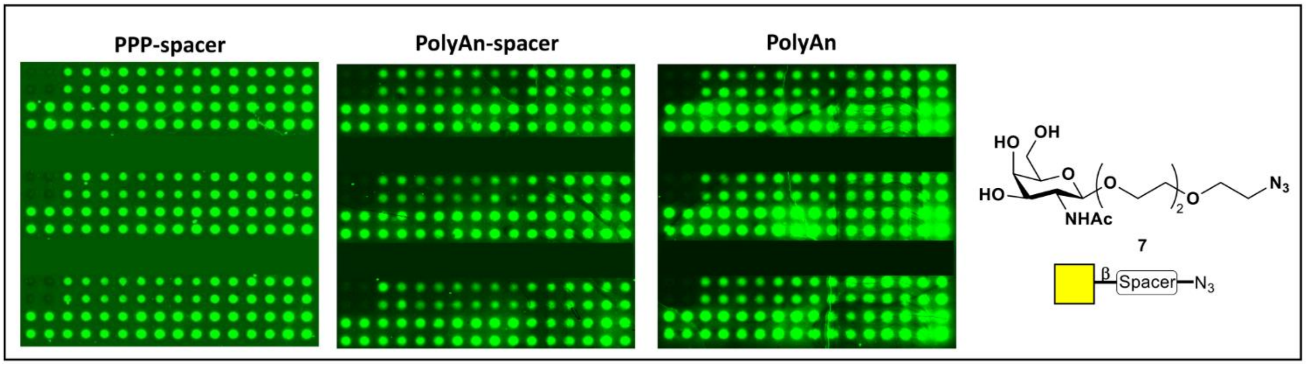

Supplementary Figure 20: Fluorescence scan image of glycopeptides containing β-GalNAc-PEG3 azide **7** after incubation with Rhodamine-labelled SBA. Staining was performed in HEPES-buffer (50 mM HEPES, 100 mM NaCl, 1 mM $CaCl_2$, 1 mM $MnCl_2$, 10 % blocking buffer and 0.05 % Tween-20, pH = 7.5) at 10 µg/mL SBA concentration. Scanning parameters: Wavelength 532 nm, PMT gain 500, laser power 33 %, pixel size 5 µm. Spot pitch is 250 µm.

Dolichos Biflorus Agglutinin (DBA)

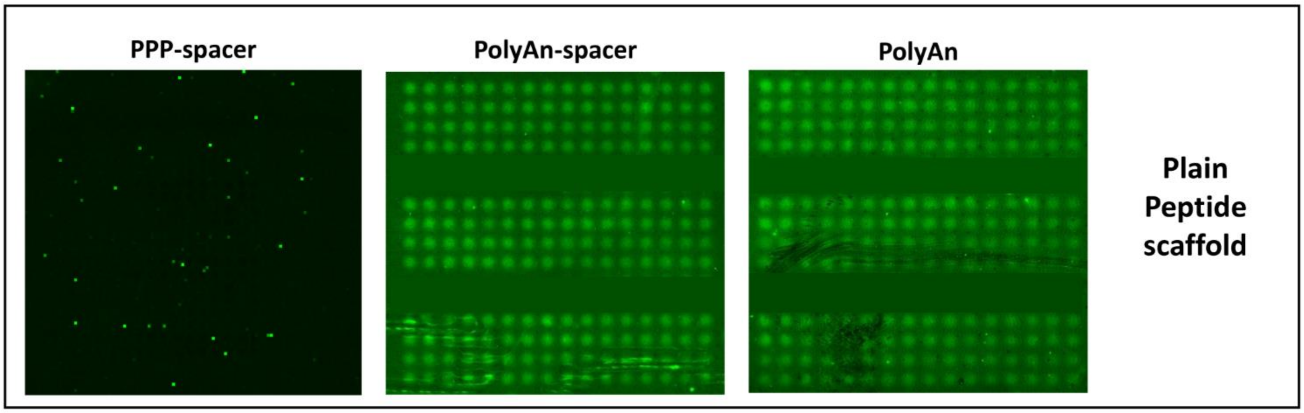

Supplementary Figure 21: Fluorescence scan image of the plain peptides (negative control) after incubation with Rhodamine-labelled DBA. Incubation was performed in HEPES-buffer (50 mM HEPES, 100 mM NaCl, 1 mM $CaCl_2$, 1 mM $MnCl_2$, 10 % blocking buffer and 0.05 % Tween-20, pH = 7.5) at 10 µg/mL lectin concentration. Scanning parameters: Wavelength 532 nm, PMT gain 500, laser power 33 %, pixel size 5 µm. Spot pitch is 250 µm**.**

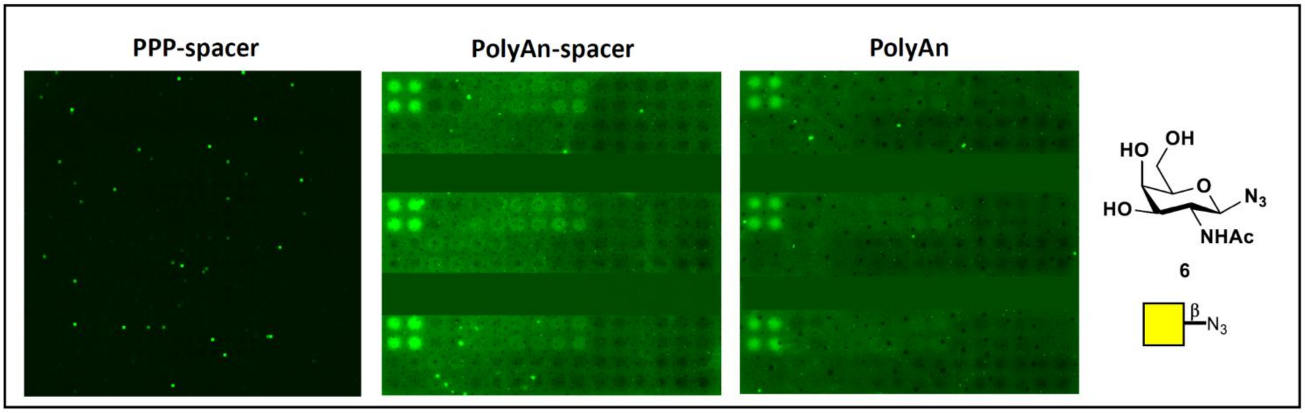

Supplementary Figure 22: Fluorescence scan image of glycopeptides containing β-GalNAc azide **6** after incubation with Rhodamine-labelled DBA. Staining was performed in HEPES-buffer (50 mM HEPES, 100 mM NaCl, 1 mM $CaCl_2$, 1 mM $MnCl_2$, 10 % blocking buffer and 0.05 % Tween-20, pH = 7.5) at 10 µg/mL DBA concentration. Scanning parameters: Wavelength 532 nm, PMT gain 500, laser power 33 %, pixel size 5 µm. Spot pitch is 250 µm.

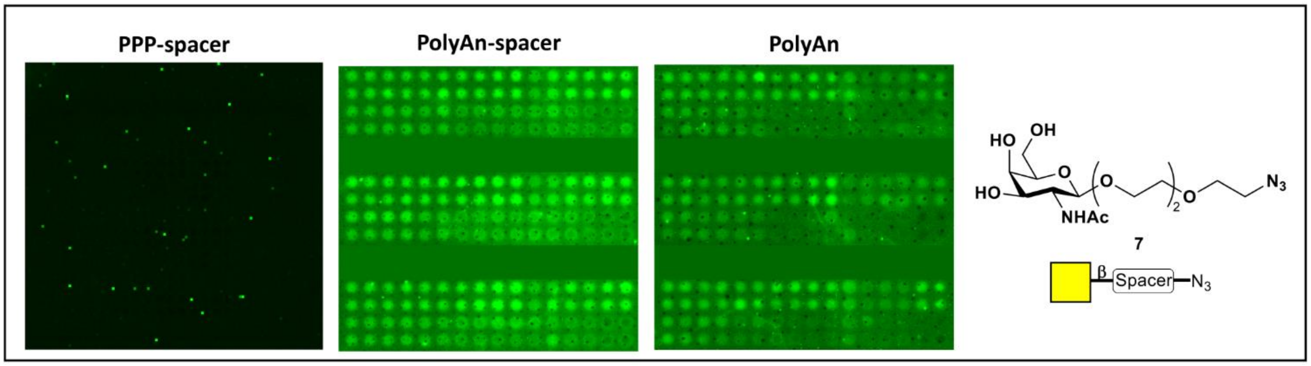

Supplementary Figure 23: Fluorescence scan image of glycopeptides containing β-GalNAc-PEG3 azide **7** after incubation with Rhodamine-labelled DBA. Staining was performed in HEPES-buffer

(50 mM HEPES, 100 mM NaCl, 1 mM $CaCl_2$, 1 mM $MnCl_2$, 10 % blocking buffer and 0.05 % Tween-20, pH = 7.5) at 10 µg/mL DBA concentration. Scanning parameters: Wavelength 532 nm, PMT gain 500, laser power 33 %, pixel size 5 µm. Spot pitch is 250 µm.

<u>Wheat Germ Agglutinin (WGA)</u>

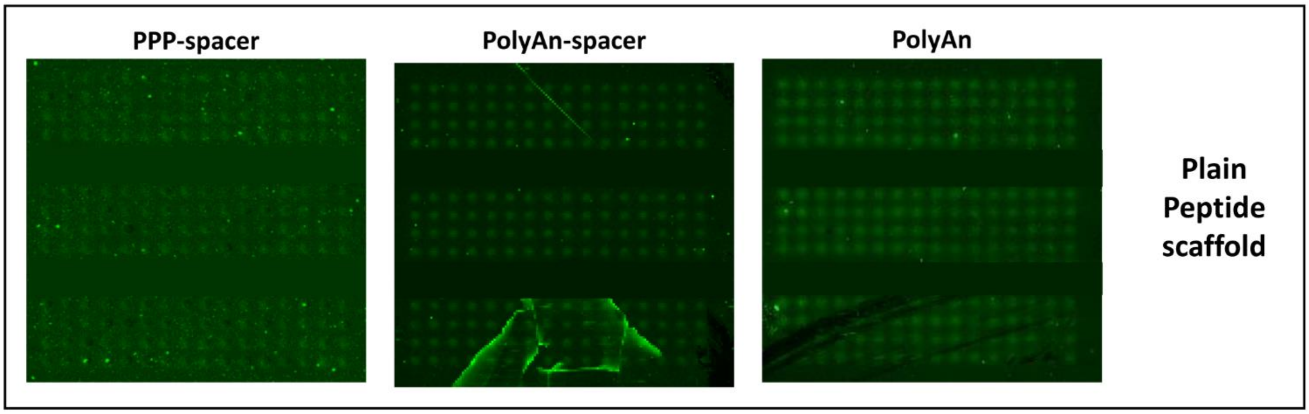

Supplementary Figure 24: Fluorescence scan image of the plain peptides (negative control) after incubation with Rhodamine-labelled WGA. Incubation was performed in HEPES-buffer (50 mM HEPES, 100 mM NaCl, 1 mM $CaCl_2$, 1 mM $MnCl_2$, 10 % blocking buffer and 0.05 % Tween-20, pH = 7.5) at 10 µg/mL lectin concentration. Scanning parameters: Wavelength 532 nm, PMT gain 500, laser power 33 %, pixel size 5 µm. Spot pitch is 250 µm.

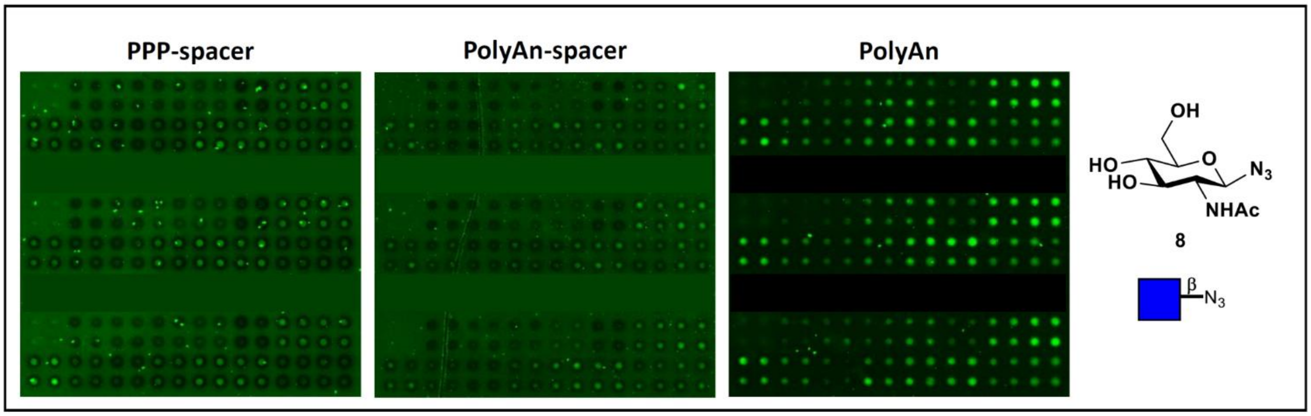

Supplementary Figure 25: Fluorescence scan image of glycopeptides containing β-GlcNAc azide **8** after incubation with Rhodamine-labelled WGA. Staining was performed in HEPES-buffer (50 mM HEPES, 100 mM NaCl, 1 mM $CaCl_2$, 1 mM $MnCl_2$, 10 % blocking buffer and 0.05 % Tween-20,

pH = 7.5) at 0.2 µg/mL WGA concentration. Scanning parameters: Wavelength 532 nm, PMT gain 500, laser power 33 %, pixel size 5 µm. Spot pitch is 250 µm.

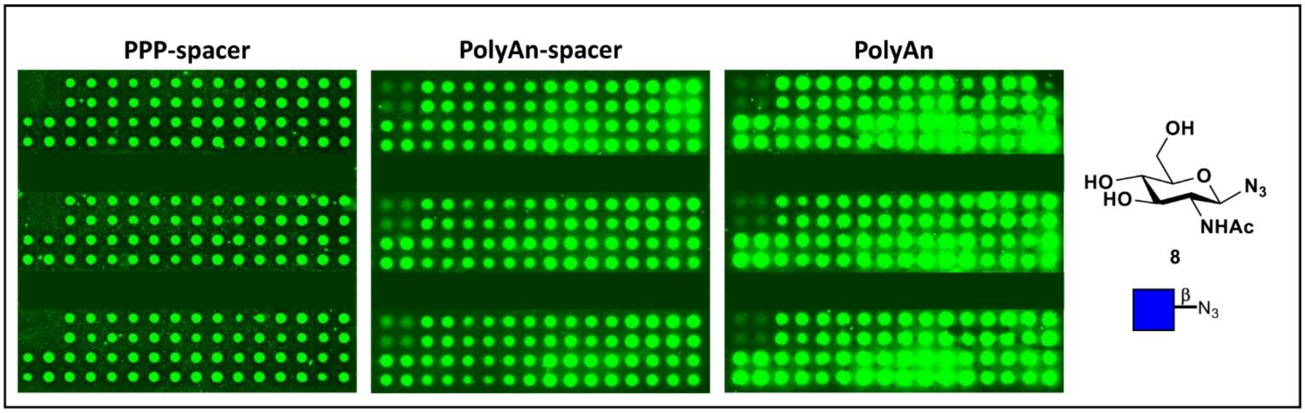

Supplementary Figure 26: Fluorescence scan image of glycopeptides containing β-GlcNAc azide **8** after incubation with Rhodamine-labelled WGA. Staining was performed in HEPES-buffer (50 mM HEPES, 100 mM NaCl, 1 mM CaCl2, 1 mM MnCl2, 10 % blocking buffer and 0.05 % Tween-20, pH = 7.5) at 10 µg/mL WGA concentration. Scanning parameters: Wavelength 532 nm, PMT gain 500, laser power 33 %, pixel size 5 µm. Spot pitch is 250 µm.

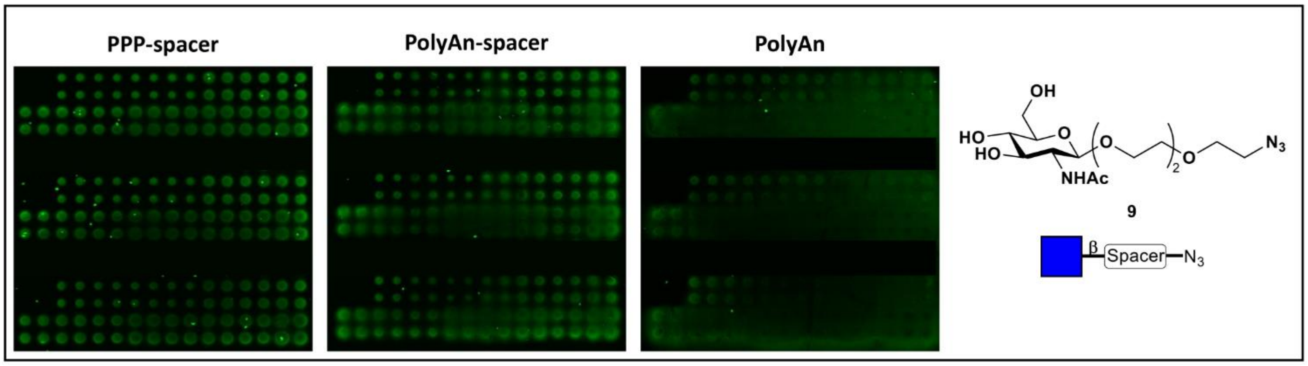

Supplementary Figure 27: Fluorescence scan image of glycopeptides containing β-GlcNAc-PEG3 azide 9 after incubation with Rhodamine-labelled WGA. Staining was performed in HEPES-buffer (50 mM HEPES, 100 mM NaCl, 1 mM $CaCl_2$, 1 mM $MnCl_2$, 10 % blocking buffer and 0.05 % Tween-20, pH = 7.5) at 0.2 µg/mL WGA concentration. Scanning parameters: Wavelength 532 nm, PMT gain 500, laser power 33 %, pixel size 5 µm. Spot pitch is 250 µm.

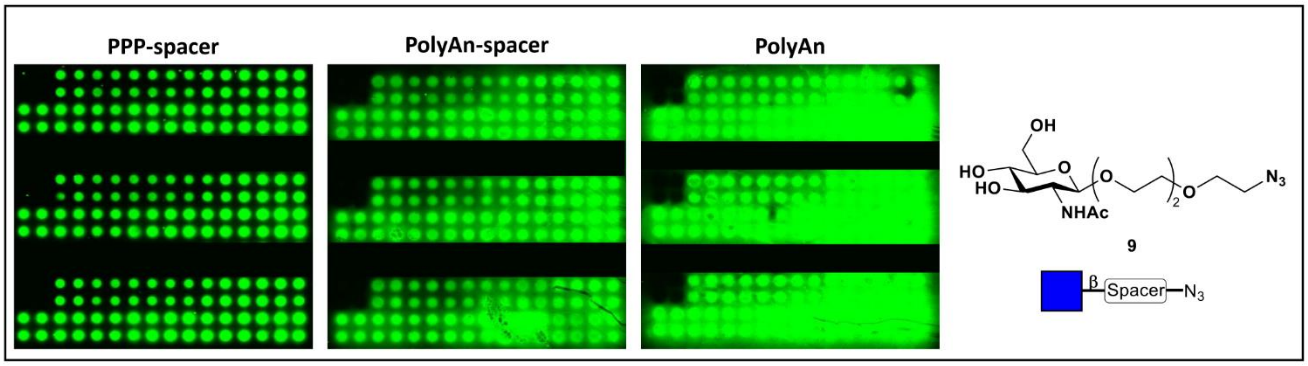

Supplementary Figure 28: Fluorescence scan image of glycopeptides containing β-GlcNAc-PEG3 azide **9** after incubation with Rhodamine-labelled WGA. Staining was performed in HEPES-buffer

(50 mM HEPES, 100 mM NaCl, 1 mM CaCl2, 1 mM MnCl2, 10 % blocking buffer and 0.05 % Tween-20, pH = 7.5) at 10 µg/mL WGA concentration. Scanning parameters: Wavelength 532 nm, PMT gain 500, laser power 33 %, pixel size 5 µm. Spot pitch is 250 µm.

10 Supporting Tables

Supplementary Table 2: Glycan-Glycan binding protein combinations tested on the microarray, table partially based on literature data.(Dessen et al., 1995; Ravishankar et al., 1997; Hamelryck et al., 1999; Wittmann and Pieters, 2013; Bartetzko et al., 2015) No binding is represented (-) while (x) the positive experimental results.

Entry	Lectin	Conc. [µg/mL]	Specificity [term. sugar]	Results		
				PPP-spacer	PolyAn-spacer	PolyAn
**1**	Concanavalin A (ConA)	100	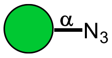	×	×	×
**2**	Ricinus communis agglutinin (RCA-I)	10	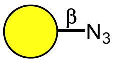	×	×	×
**3**	Ricinus communis agglutinin (RCA-I)	10	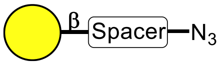	×	×	×
**4**	Ricinus communis agglutinin (RCA-I)	10	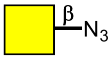	no binding (not shown)	-	-
**5**	Ricinus communis agglutinin (RCA-I)	10	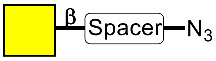	no binding (not shown)	-	-
**6**	Peanut agglutinin (PNA)	10	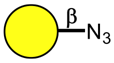	×	×	×
**7**	Peanut agglutinin (PNA)	10	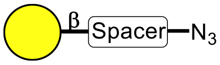	×	×	×
**8**	Peanut agglutinin (PNA)	100	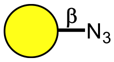	×	×	×
**9**	Peanut agglutinin (PNA)	100	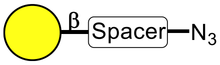	×	×	×
**10**	Peanut agglutinin (PNA)	100	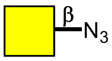	No binding, (not shown)	-	-

**11**	Soybean agglutinin (SBA)	10	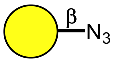	No binding (not shown)		
**12**	Soybean agglutinin (SBA)	10	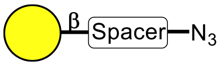	No binding (not shown)		
**13**	Soybean agglutinin (SBA)	10	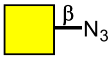	No binding (not shown)		
**14**	Soybean agglutinin (SBA)	20	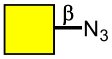	No binding (not shown)	-	-
**15**	Soybean agglutinin (SBA)	10	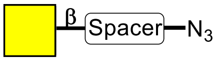	×	×	×
**16**	Dolichos biflorus agglutinin (DBA)	10	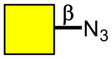	No binding	×	×
**17**	Dolichos biflorus agglutinin (DBA)	20	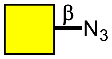	No binding (not shown)	-	-
**18**	Dolichos biflorus agglutinin (DBA)	10	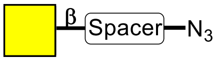	No binding	×	×
**19**	Wheat germ agglutinin (WGA)	0.2	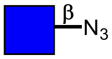	×	×	×
**20**	Wheat germ agglutinin (WGA)	10	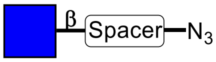	×	×	×
**21**	Wheat germ agglutinin (WGA)	10	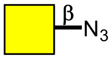	No binding (not shown)	-	-

11 References

Artigas, G., Monteiro, T., Hinou, H., Nishimura, S.-I., Lepenies, B., and Garcia-Martin, F. (2017). Glycopeptides as Targets for Dendritic Cells: Exploring MUC1 Glycopeptides Binding Profile toward Macrophage Galactose-Type Lectin (MGL) Orthologs. doi:10.1021/acs.jmedchem.7b01242.

Bartetzko, M. P., Schuhmacher, F., Hahm, H. S., Seeberger, P. H., and Pfrengle, F. (2015). Automated Glycan Assembly of Oligosaccharides Related to Arabinogalactan Proteins. *Org. Lett.* 17, 4344–4347. doi:10.1021/acs.orglett.5b02185.

Dam, T. K., and Brewer, C. F. (2010). Lectins as pattern recognition molecules: The effects of epitope density in innate immunity *. *Glycobiology* 20, 270–279. doi:10.1093/glycob/cwp186.

Dessen, A., Gupta, D., Sabesan, S., Brewer, C. F., and Sacchettini, J. C. (1995). X-ray Crystal Structure of the Soybean Agglutinin Cross-Linked with a Biantennary Analog of the Blood Group I Carbohydrate Antigen. *Biochemistry* 34, 4933–4942. doi:10.1021/bi00015a004.

Di Maio, A., Cioce, A., Achilli, S., Thépaut, M., Vivès, C., Fieschi, F., et al. (2021). Controlled density glycodendron microarrays for studying carbohydrate–lectin interactions. *Org. Biomol. Chem.* doi:10.1039/D1OB00872B.

Hamelryck, T. W., Loris, R., Bouckaert, J., Dao-Thi, M. H., Strecker, G., Imberty, A., et al. (1999). Carbohydrate binding, quaternary structure and a novel hydrophobic binding site in two legume lectin oligomers from Dolichos biflorus. *J. Mol. Biol.* 286, 1161–1177. doi:10.1006/jmbi.1998.2534.

Kang, B., Okwieka, P., Schöttler, S., Winzen, S., Langhanki, J., Mohr, K., et al. (2015). Carbohydrate-Based Nanocarriers Exhibiting Specific Cell Targeting with Minimum Influence from the Protein Corona. *Angew. Chemie - Int. Ed.* 54, 7436–7440. doi:10.1002/anie.201502398.

Karpitschka, S., Liebig, F., and Riegler, H. (2017). Marangoni Contraction of Evaporating Sessile Droplets of Binary Mixtures. *Langmuir* 33, 4682–4687. doi:10.1021/acs.langmuir.7b00740.

Kong, N., Shimpi, M. R., Park, J. H., Ramström, O., and Yan, M. (2015). Carbohydrate conjugation through microwave-assisted functionalization of single-walled carbon nanotubes using perfluorophenyl azides. *Carbohydr. Res.* 405, 33–38. doi:10.1016/j.carres.2014.09.006.

Loris, R., Hamelryck, T., Bouckaert, J., and Wyns, L. (1998). Legume lectin structure. *Biochim. Biophys. Acta - Protein Struct. Mol. Enzymol.* 1383, 9–36. doi:10.1016/S0167-4838(97)00182-9.

Maglinao, M., Eriksson, M., Schlegel, M. K., Zimmermann, S., Johannssen, T., Götze, S., et al. (2014). A platform to screen for C-type lectin receptor-binding carbohydrates and their potential for cell-specific targeting and immune modulation. *J. Control. Release* 175, 36–42. doi:10.1016/j.jconrel.2013.12.011.

Mayer, S., Raulf, M.-K., and Lepenies, B. (2017). C-type lectins: their network and roles in pathogen recognition and immunity. *Histochem. Cell Biol.* 147, 223–237. doi:10.1007/s00418-016-1523-7.

Mende, M., Tsouka, A., Heidepriem, J., Paris, G., Mattes, D. S., Eickelmann, S., et al. (2020). On-Chip Neo-Glycopeptide Synthesis for Multivalent Glycan Presentation. *Chem. – A Eur. J.* 26, 9954–9963. doi:10.1002/chem.202001291.

Natchiar, S. K., Suguna, K., Surolia, A., and Vijayan, M. (2007). Peanut agglutinin, a lectin with an unusual quaternary structure and interesting ligand binding properties. *Crystallogr. Rev.* 13, 3–28. doi:10.1080/08893110701382087.

Pastuch-Gawołek, G., Malarz, K., Mrozek-Wilczkiewicz, A., Musioł, M., Serda, M., Czaplinska, B., et al. (2016). Small molecule glycoconjugates with anticancer activity. *Eur. J. Med. Chem.* 112, 130–144. doi:10.1016/j.ejmech.2016.01.061.

Pereira, M. E. A., Kabat, E. A., and Sharon, N. (1974). Immunochemical studies on the specificity of soybean agglutinin. *Carbohydr. Res.* 37, 89–102. doi:10.1016/S0008-6215(00)87066-4.

Ravishankar, R., Ravindran, M., Suguna, K., Surolia, A., and Vijayan, M. (1997). Crystal structure of the peanut lectin - T-antigen complex. Carbohydrate specificity generated by water bridges. *Curr. Sci.* 72, 855–861. doi:10.2210/pdb2tep/pdb.

Shiba, A., Kinoshita-Kikuta, E., Kinoshita, E., and Koike, T. (2017). TAMRA/TAMRA Fluorescence Quenching Systems for the Activity Assay of Alkaline Phosphatase. *Sensors* 17, 1877. doi:10.3390/s17081877.

Stadler, V., Kirmse, R., Beyer, M., Breitling, F., Ludwig, T., and Bischoff, F. R. (2008). PEGMA/MMA Copolymer Graftings: Generation, Protein Resistance, and a Hydrophobic Domain. *Langmuir* 24, 8151–8157. doi:10.1021/la800772m.

Terada, Y., Hoshino, Y., and Miura, Y. (2019). Glycopolymers Mimicking GM1 Gangliosides: Cooperativity of Galactose and Neuraminic Acid for Cholera Toxin Recognition. *Chem. – An Asian J.* 14, 1021–1027. doi:10.1002/asia.201900053.

Tsoulougian, V., Psykarakis, E. E., and Gimisis, T. (2019). Synthesis of biurets via TMSNCO addition to 1-aminosugars: application in the de novo synthesis of dC oxidation products. *Org. Biomol. Chem.* 17, 973–981. doi:10.1039/C8OB02810A.

Valverde, P., Daniel Martínez, J., Cañada, F. J., Ardá, A., and Jiménez-Barbero, J. (2020). Molecular

Recognition in C-Type Lectins: The Cases of DC-SIGN, Langerin, MGL, and L-Sectin ChemBioChem. *ChemBioChem* 21, 2999–3025. doi:10.1002/cbic.202000238.

Wittmann, V., and Pieters, R. J. (2013). Bridging lectin binding sites by multivalent carbohydrates. *Chem. Soc. Rev.* 42, 4492. doi:10.1039/c3cs60089k.
